# Whole Fruits and Fruit Fiber Emerging Health Effects

**DOI:** 10.3390/nu10121833

**Published:** 2018-11-28

**Authors:** Mark L. Dreher

**Affiliations:** Nutrition Science Solutions, LLC, Wimberley, 78676 TX, USA; mdreher@nutriscisolutions.com or nss3@sbcglobal.net; Tel.: +1-512-847-9182

**Keywords:** prebiotic effects, gastrointestinal health, weight management, cardiovascular disease, diabetes, metabolic syndrome, successful aging, cancer, psychological well-being, depression, asthma, bone mineral density

## Abstract

Less than 10% of most Western populations consume adequate levels of whole fruits and dietary fiber with typical intake being about half of the recommended levels. Evidence of the beneficial health effects of consuming adequate levels of whole fruits has been steadily growing, especially regarding their bioactive fiber prebiotic effects and role in improved weight control, wellness and healthy aging. The primary aim of this narrative review article is to examine the increasing number of health benefits which are associated with the adequate intake of whole fruits, especially fruit fiber, throughout the human lifecycle. These potential health benefits include: protecting colonic gastrointestinal health (e.g., constipation, irritable bowel syndrome, inflammatory bowel diseases, and diverticular disease); promoting long-term weight management; reducing risk of cardiovascular disease, type 2 diabetes and metabolic syndrome; defending against colorectal and lung cancers; improving odds of successful aging; reducing the severity of asthma and chronic obstructive pulmonary disease; enhancing psychological well-being and lowering the risk of depression; contributing to higher bone mineral density in children and adults; reducing risk of seborrheic dermatitis; and helping to attenuate autism spectrum disorder severity. Low whole fruit intake represents a potentially more serious global population health threat than previously recognized, especially in light of the emerging research on whole fruit and fruit fiber health benefits.

## 1. Introduction

The beneficial health effects of consuming healthy dietary patterns rich in dietary fiber from whole plant foods include: improving gut health; lowering elevated LDL-cholesterol; reducing the risk of excessive weight gain and obesity; decreasing cardiovascular disease (CVD), coronary heart disease (CHD) and mortality risks; reducing risks of several cancers, stroke and type 2 diabetes; and improving the odds for successful aging [1,2,3,4,5]. Whole fruits (e.g., fresh, frozen, canned or dried) are recognized for their fiber content, very low to moderate energy density, and as being important sources of healthy nutrients (e.g., potassium and vitamin C) and phytochemicals (e.g., polyphenols and carotenoids), which work synergistically to support a wide range of health benefits [1,2,6,7,8,9]. Although most of the fiber from whole fruit is removed during fruit juice processing, 100% fruit juices retain similar levels of other healthy vitamins, minerals and phytochemicals [10]. About 90% of the US (and other Western) populations do not eat the recommended daily intake of fruit (e.g., 1 to 1 ½ cups for children 2–8 years, 1 ½ cups for adolescent girls and boys, 1 ½ to 2 cups for women, and 2 cups for men) [11]. The typical daily fruit intake is about half the recommended level with juice consumption making up one-third of this level for adults and one-half for children [11]. Consequently, fruit fiber is a relatively small component of the total fiber consumed in populations eating a Western dietary pattern.

**Figure 1:**
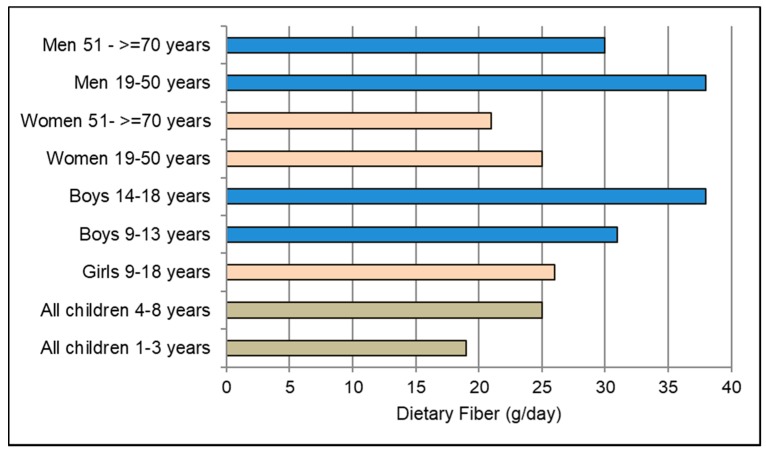
Recommended daily dietary fiber intake by age and gender [18].

The 2015–2020 Dietary Guidelines for Americans named fiber as a major shortfall nutrient of important public health concern [1,12]. Fiber is defined as carbohydrates from plant cell walls, resistant starch and oligosaccharides that are resistant to gastric acidity, hydrolysis by mammalian enzymes, and absorption in the upper gastrointestinal tract. The human gastrointestinal tract, and cardiometabolic and immune systems evolved on high fiber plant-based diets (≥50 g total fiber/day), including the consumption of wild berries and other native fiber-rich edible plants by hunter-gathers, and later grains, fruits and vegetables from traditional farming, which provided fiber rich diets until the mass globalization of Western dietary patterns in the 20th and 21st centuries. The low fiber Western diet has contributed to increased risk of weight gain, inflammation, chronic diseases and other health concerns in large part by increasing the risk of colonic microbiota dysbiosis associated with unhealthy immunity, cardiometabolic and energy regulatory processes [13,14,15,16,17]. With the current high prevalence of the Western diet, only about 3% of men and 6% of women habitually consume ≥14 g fiber/1000 kcal, the threshold level considered adequate for optimal health [18,19,20,21,22,23,24]. In most Western countries, the typical fiber intake is about half the adequate level. Specific adequate total daily fiber intake varying by age and gender is summarized in Figure 1 [18]. Fiber-rich, plant-based dietary patterns are a major contributor to prebiotic health effects, which stimulate the growth of beneficial intestinal bacterial species to promote and maintain a healthy colonic microbiota ecosystem (microbiome) in large part from fermentation of fiber to short chain fatty acids (SCFAs) [1,2,3,4,9,14,15,16,17,22,23,24,25,26,27,28,29,30,31,32,33,34,35]. 

The primary aim of this narrative review article is to examine emerging prebiotic and other health effects associated the intake of whole fruits, especially fruit fiber, throughout the human lifecycle. 

## 2. Fruit as a Prebiotic Source

### 2.1. Fruit Fiber Components and Fermentability

A comprehensive table of whole fruit total fiber and sub-components, sugar, and energy content per serving is provided in Table 1. As fruit ripens after harvest, the 3-dimensional hydrated cell wall fiber components pectin, hemicellulose and cellulose become increasingly disassembled, allowing for more microbial access and enhancing susceptibility to fermentation [26,27,28,36,37]. The process of eating, and then digestion in the upper gastrointestinal tract further breaks down the whole fruit into smaller particles and damages the fruit cell wall surfaces to enable greater colonic bacterial enzymatic breakdown and fermentation. Starchy fruits (e.g., bananas and plantains) provide fiber from both cell walls and resistant starch stored within the cells [32,38]. Generally, it has been assumed that soluble fibers are fermented more rapidly and completely compared with insoluble fibers, but this view is changing, especially concerning ripe fresh fruits, with cell wall “insoluble fibers” in a hybrid hydrated and partially disassembled state with increased susceptibility to fermentation [22,26]. Also, it is hard to rely on insoluble and soluble quantifications as current standardized methods have many limitations in accurately separating them into individual fractions. Whole fruit can provide a major source of fermentable fiber to support colon prebiotic activity, which can contribute to a wide range of potential human health benefits with sustained consumption at recommended levels. 

### 2.2. “In Vitro” Human Colonic Microbiota Model System Studies

#### 2.2.1. Pectin

The most extensively studied fruit fiber prebiotic component is pectin, which comprises on average 35% of fruit fiber cell wall content [9,37]. A Korean in vitro human colonic microbiota model system study (3 male donors with diverse microbiota profiles) confirmed the effectiveness of pectin in promoting robust prebiotic activity in all subjects [39]. Although donors had differences in baseline colonic microbiota composition, there were similar increases in healthy bacteria after pectin fermentation including species belonging to butyrate producing *Clostridium* cluster XIV (e.g., *Lachnospira*), and *Sutterella*. After pectin intake, microbiota production of SCFAs, acetate and butyrate levels increased 6 h post intake. Acetate continuously increased up to 18 h, then rapidly decreased by 36 h. Butyrate steadily increased approximately 28% by 48 h. Propionate slowly increased after 12 h until about 48 h at a much slower rate than acetate or butyrate. Increased butyrate levels are used as the main energy source by colonocytes to maintain the colonic protective barrier and in lowering colonic luminal pH to inhibit pathogenic bacteria. A comparison between apple pectin and inulin using a human colonic microbial anaerobic continuous-flow fermenter found that they were both effective in promoting prebiotic activity with anti-inflammatory effects, although apple pectin was three times more effective in promoting *Bacteroides* and overall microflora diversity than inulin, presumably reflecting the differing complexity of the two prebiotics [40]. A recent study using an in vitro Simulator of the Human Intestinal Microbial Ecosystem (SHIME^®^) suggests that citrus pectin stimulated the production of butyric acid in the simulated transverse and descending colon, as well as the growth of genera *Lactobacillus*, *Megamonas*, and *Lachnospiracea* with related anti-inflammatory effects, and a reduction of ammonium ions [41]. A 2018 in vitro study also suggests that pectins have the potential to improve the survival of probiotic bacteria such as *Lactobacillus* in the stomach and small intestine [42]. Overall, these studies find that fruit fiber, especially pectin, can help re-balance the colonic microbiota towards a higher anti-inflammatory profile by: (1) increasing the *Bacteroidetes/Firmicutes* ratio, and increasing the abundance of *Bifidobacterium and Clostridium* cluster XIV, resulting in enhanced colonic mucosal barrier integrity and function, increased mucosal immunity, increased butyrate production, and a decrease in enteric pathogens; (2) promoting *Eubacterium eligens*, which upregulates pectinolytic enzymes; and (3) supporting certain *Faecalibacterium prausnitzii* strains in utilizing the fermentation of pectin to exert anti-inflammatory effects [31,32,33,43,44,45,46,47,48]. Pectin is a major fruit prebiotic that has been extensively studied and shown to promote a healthy, anti-inflammatory colonic microbiota ecosystem with greater microflora diversity than inulin.

#### 2.2.2. Fruits

Several in vitro human fecal microbiota model fermentation systems studies support the prebiotic effects of whole fruits. A study which evaluated apples with an in vitro batch culture colonic model system (pH 5.5–6.0; 37 °C; inoculated with feces from three healthy donors; 3 varieties) found that all the apple varieties exhibited beneficial prebiotic activity by improving colonic microbiota bacterial diversity, and increasing *Actinobacteria* relative abundance and total SCFAs levels (*p* < 0.05) [49]. The Renetta Canada apple variety, rich in fiber and polyphenols in particular had positive consequences for human health by increasing *Bifidobacteria*, *F. prausnitzii*, butyrate levels and polyphenol microbial metabolites (*p* < 0.05). Raisins assessed with a simulated in vitro digestive system with dynamic mastication, gastric, duodenal and colonic human models showed that raisins significantly increased the proportion of *Bifidobacteria* and *Lactobacilli*, decreased numbers of *Firmicutes*, and approximately doubled the fecal concentration of propionate and butyrate compared to controls [50]. Commonly consumed whole fruits can promote a healthy microbiota ecosystem by the actions of their fiber and polyphenolics. 

### 2.3. Human Trials

**Figure 2:**
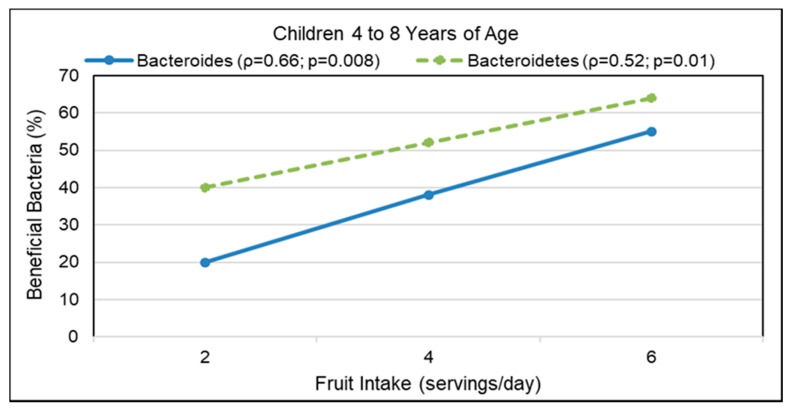
Correlations between daily servings of fruit intake and the microbiota abundance of beneficial *Bacteroides* and *Bacteroidetes* in children [51].

A growing number of human trials support the prebiotic effects of whole fruit and fruit fiber in promoting a healthy microbiome. A 2016 dose-response Randomized Controlled Trial (RCT) (122 subjects; 2, 4 and 6 fruit and vegetable servings vs. a habitual control diet) found that the fiber content of fruits and vegetables was more important than the polyphenol content in promoting healthier fecal microflora (e.g., *Bacteroide/Prevotella* ratio and *Bifidobacteria)* [35]. A longitudinal study (22 children; age 4 to 8 years) showed that the number of fruit servings/day was positively correlated with the relative abundance of colonic bacteria *Bacteroides* and *Bacteroidetes* in the children studied (Figure 2), suggesting that higher fruit fiber intake is associated with a healthier microbiome for better childhood immune function compared to the typical low fruit intake by children in the US [51]. A growing number of human studies on specific fruit sources show varying levels of prebiotic effects depending on the fruit source and daily amount consumed when compared to control low fiber diets or food. For apples, an open trial (8 healthy men; 2 apples/day added to usual diet vs. baseline) showed that apples significantly increased the proportion of *Bifidobacteria* in feces on day 7 (*p* < 0.05) and day 14 (*p* < 0.01), decreased fecal *C. perfringens, Enterobacteriaceae* and *Pseudomonas* with a trend toward higher levels of fecal SCFAs [52]. For prunes, a dose-response parallel RCT (120 constipated adults; low fiber diets) found that ≥80 g prunes [6 g fiber]/d significantly increased *Bifidobacteria* and stool weight and frequency with trends for higher SCFAs levels and lower stool pH [53]. For bananas, four RCTs in adults and children found that bananas and banana flakes were significantly more effective in reducing chronic pathogenic bacterial diarrhea severity (e.g., severe *Shigellosis* dysentery, *Clostridium difficile*) compared to control food, rice-based diets or standard medical treatment [54,55,56,57]. For plantains, a RCT (80 infants and children with persistent pathogenic bacterial diarrhea) showed that cooked plantains significantly shortened average duration of diarrhea by 18 h compared to yogurt [58]. For kiwi fruit, an open trial (6 young Chinese women) found that 2 kiwi fruit equivalents/day enhanced growth of lactic acid bacteria and decreased levels of pathogenic bacteria within 24 h compared to diets restricted in fruit and yogurt [59]. For dates, a crossover RCT (22 healthy men and women; 7 dates or 50 g vs. refined carbohydrate control) demonstrated that a single serving of dates produced a non-significant increase of healthy microbiota bacterial groups and SCFAs, but beneficial changes were shown for stool frequency, ammonia concentrations and genotoxicity of fecal water [60]. Generally, at least 2 daily whole fruit servings, especially fruits with ≥2.5 g fiber/serving are required to stimulate significant colonic prebiotic activity (higher SCFAs, production, increased levels of *Bifidobacteria*, reduced levels of pathogenic bacteria and protection against persistent bacterial diarrhea in children and adults) compared to ≤1 whole fruit serving/day common in Western diets [1,2,11,12]. 

## 3. Emerging Health Benefits Associated with Whole Fruits and Fruit Fiber

### 3.1. Gastrointestinal Tract

Acute or chronic colonic gastrointestinal health issues affect the well-being of all humans with variations depending on age, genetic propensity, food borne pathogenic infections, diet and water quality, level of fluid intake, physical activity, medications, stress coping ability and other factors. 

#### 3.1.1. Constipation

The prevalence of constipation worldwide is approximately 16% in all adults and 33.5% in adults over 60 years [61]. The risk of constipation is affected by genetic predisposition, socioeconomic status, low fiber intake, inadequate fluid intake, lack of mobility, hormone imbalance, and side effects of medications. A RCT (80 Chinese adults with slow-transit constipation) found that pectin significantly reduced mean constipation score and increased the population of colonic healthy microflora [62]. A crossover RCT showed that increased intake of whole fruits and vegetables significantly reduced fecal transit time by 14 h, increased the number of daily bowel movements by 0.4, and daily wet fecal weight by 118 g compared to 100% fruit and vegetable juices [63]. The Bristol Stool Scale may be useful as a quick indicator of microbiota health quality since the stool quality shape rating and increased fecal mass (about 50% bacteria) are crude indicators of microbiome health [22]. The viscous, low to moderate fermentable soluble fiber in whole fruits can effectively relieve constipation symptoms. For mangos, a parallel RCT showed that 300 g (2 servings)/day of fresh mangos, approximately 5 g of mango fiber, significantly reduced total constipation score (including frequency of bowel movements, difficulty/straining to evacuate, pain on evacuation, abdominal pain, daily evacuation attempts, and duration of constipation) by 60% in chronically constipated adults compared to baseline [64]. For prunes, a systematic review (4 RCTs) and a RCT found that in constipated subjects, 2 daily servings of prunes were as effective as 2 daily servings of psyllium for increasing stool frequency and improving stool consistency [65,66]. For kiwi fruit, twice daily intake improved laxation, softened stools, and relieved chronic constipation with the active component being a highly viscous soluble fiber with modest fermentability [67,68]. The daily intake of 2 servings of fiber rich fruits can promote regularity and help to protect against constipation.

#### 3.1.2. Irritable Bowel Syndrome

Irritable bowel syndrome (IBS), characterized by altered bowel motility, abdominal pain, flatulence, and abnormal immune function, is the most common chronic functional gastrointestinal 

disorder. It affects about 10–15% of the global population and is most prevalent in people <45 years [69]. A cohort study observed that IBS patients have significantly lower microbial diversity, butyrate-producing bacteria and *Methanobacteria* compared to healthy controls. This is associated with colonic dysbiosis, reduced intestinal barrier function and impaired colonic hydrogen disposal linked to an excess of abdominal gas [70]. Although short chain highly fermentable prebiotic supplements should be restricted to avoid the risk of a rapid increase in gas, bloating/distension, and abdominal pain/discomfort, the consumption of long-chain or complex, soluble and more slowly fermentable, butyrate producing fibers, such as fruit pectin may have a role in helping to control IBS symptoms [71,72]. A Chinese RCT (IBS diarrhea) found that pectin significantly increased *Bifidobacteria*, decreased total *Clostridium* sp (*p* < 0.05), reduced IBS symptoms, normalized cytokine levels, and improved Bristol stool scale score and quality of life assessments [73]. Another RCT (IBS-constipation) showed that 2 kiwi fruit/day significantly shortened colon transit time, increased defecation frequency and improved overall bowel function [74]. Two servings/day of low FODMAP (fermentable oligosaccharides, disaccharides, monosaccharides and polyols) fresh fruit including: bananas, blueberries, oranges, durians, grapefruits, grapes, honeydew melons, avocados, kiwifruit, lemons, limes, mandarin oranges, passion fruit, raspberries, and strawberries, have been proposed as a potential dietary option for IBS management, allowing 2–3 h between each fruit serving to avoid over loading the gastrointestinal system and to confirm the effectiveness of any specific fruit [75]. The daily intake of 2–3 servings/day of low FODMAP fruits or 5–10 g/day fruit fiber may help to reduce IBS risk and prevent flare-up in IBS patients. 

#### 3.1.3. Inflammatory Bowel Diseases

Inflammatory bowel disease (IBD) is a chronic inflammatory condition including Crohn’s disease (CD) and ulcerative colitis (UC) with active clinical symptoms including diarrhea and/or constipation, stool containing mucus and/or blood and varying degrees of abdominal cramping followed by periods of remission [76]. Although healthy diets including high fiber and low FODMAP fruits and vegetables have been recommended to reduce IBD pathogenesis and prolong maintenance of clinical remission, more well-designed studies on the effects of diet are needed to help dietitians to provide better evidence-based nutritional guidance to IBD patients. IBD risk may be associated with low fiber diets, which are related to a dysfunctional microbiome and reduced microbiota fecal concentrations of butyrate. The adequate intake of prebiotic fiber can increase colonic butyrate to levels needed to maintain the mucosal barrier function to protect against IBD inflammatory pathogenesis [77]. 

##### Crohn’s Disease (CD)

**Figure 3:**
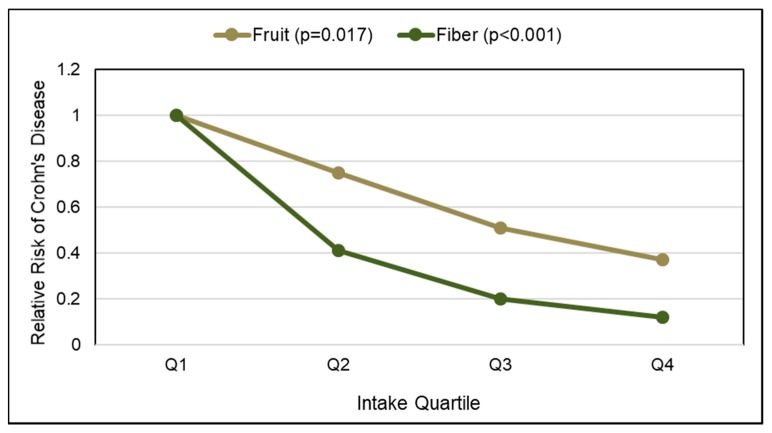
Risks of Crohn’s disease in children for fruit and total fiber intake [82].

CD is a transmural inflammatory condition that can affect both the large and small intestine, and may be associated with bowel obstruction, strictures, and/or abscesses [76]. There are two identified peaks for occurrence of CD: (1) age range of teens to 35 years and (2) 60–80 years [78]. A meta-analysis (10 case-control studies) [79], a systematic review (5 cohort or case-control studies) [80], and The Nurses’ Health Study [81] found that increased fruit intake reduced CD risk in these populations by approximately 40%. Conversely, increased vegetable and whole grain consumption was not significantly associated with lower CD risk [79,80,81]. A 2007 Canadian case-controlled study in children found significant reduction in CD risk for higher fruit and total fiber intake (Figure 3), suggesting that increased fruit fiber may have a role in reducing CD risk [82]. A 2015 meta-analysis (7 cohort and case-control studies) observed a linear dose-response decrease in CD risk by 13% for each 10 g/day total fiber increment (*p* < 0.05), which is equivalent to 2 to 3 whole fruit servings/day [83]. A-2016 internet cohort study (1619 adults with CD remission) observed that participants who reported that they did not avoid fiber rich foods such as whole fruits were 40% less likely to have a CD flare-up than those who avoided high fiber foods [84]. However, a 2018 European prospective multi-center cohort (EPIC-IBD) study did not observe any statistically significant associations between total fiber or fiber from fruit, vegetables or cereals with reduced CD risk [85]. Although the adequate intake of whole fruit and fruit fiber may help to reduce CD risk and prolong CD remission periods, studies on the specific effects of fruit fiber on CD risk are limited. 

##### Ulcerative Colitis (UC)

UC is a non-transmural inflammatory condition which only affects the colon mucosa and superficial submucosa in adults [76]. A meta-analysis (9 case-control studies) observed that higher consumption of fruits and vegetables was associated with an approximately 30% reduction in UC risk [79]. A systematic review (8 cohort and case-control studies) observed that fruit intake was not significantly associated with lower UC risk but high vegetable intake was associated with decreased risk of UC [80]. A 2018 EPIC-IBD study did not observe any statistically significant associations between total fiber or fiber from fruit, vegetables or cereals with reduced UC risk [85]. There is limited and inconsistent evidence supporting beneficial effects of fruit and fruit fiber in reducing UC risk or prolonging UC remission. 

#### 3.1.4. Diverticular Disease 

Colonic diverticulosis, characterized by herniation or out-pouching of the colon known as diverticula, is progressively more prevalent with aging, occurring in about 30% of people at age 60 years and 70% of people ≥80 years [86]. However, only 10–25% of individuals with diverticulosis develop symptoms such as abdominal pain and changes in bowel habit and 4% have a lifetime risk of developing serious life-threatening complications with high medical and hospitalization costs. Risk factors associated with diverticular disease include obesity, and diets low in fiber and high in red meat [87]. A cross-sectional study observed that patients with diverticular disease had colonic microbiota dysbiosis including increased colonic inflammation associated with increased presence of macrophages [88]. The Swedish Mammography Cohort and the Cohort of Swedish Men (approx. 89,000 middle-age and elderly women and men) observed that those with the highest intake of fruit and vegetable fibers had an approximately 30% decreased risk of hospitalization compared to those with the lowest intake but cereal fiber did not affect risk [89]. The UK Million Women Study (approximately 700,000 women) observed that diverticular disease risk was significantly reduced per 5 g of fruit fiber by 19% and 5 g of cereal fiber by 16% compared to no risk reduction effects for either vegetable or potato fiber [90]. A review of diet and diverticular disease suggests that increased fruit fiber intake reduced diverticular disease risk by 38% [91]. An EPIC UK/Oxford cohort (47,033 adults) observed that higher total fiber intake (≥26 g/day vs. <14 g/day) was associated with a significantly reduced risk of diverticular disease by 41% and vegetarian diets were significantly associated with a 31% lower risk of diverticular disease compared to diets with high meat intake [92]. A 2018 systematic review concluded that the intake of adequate fiber after an acute episode of symptomatic uncomplicated diverticular disease can reduce the risk of future flare-ups [93]. Adequate fruit and fiber intake appear to be effective in reducing diverticular disease risk and flare-ups or complications including hospitalizations. 

### 3.2. Weight Control

The growing worldwide overweight and obesity pandemic is among the greatest public health challenges of our time affecting approximately 2 billion plus people worldwide. Its causes are multifactorial involving unhealthy lifestyle factors including the consumption of energy dense and low fiber Western diets combined with sedentary lower energy expenditure, genetic, environmental, and emotional factors. 

#### 3.2.1. Observational Studies

##### Fruit

**Figure 4:**
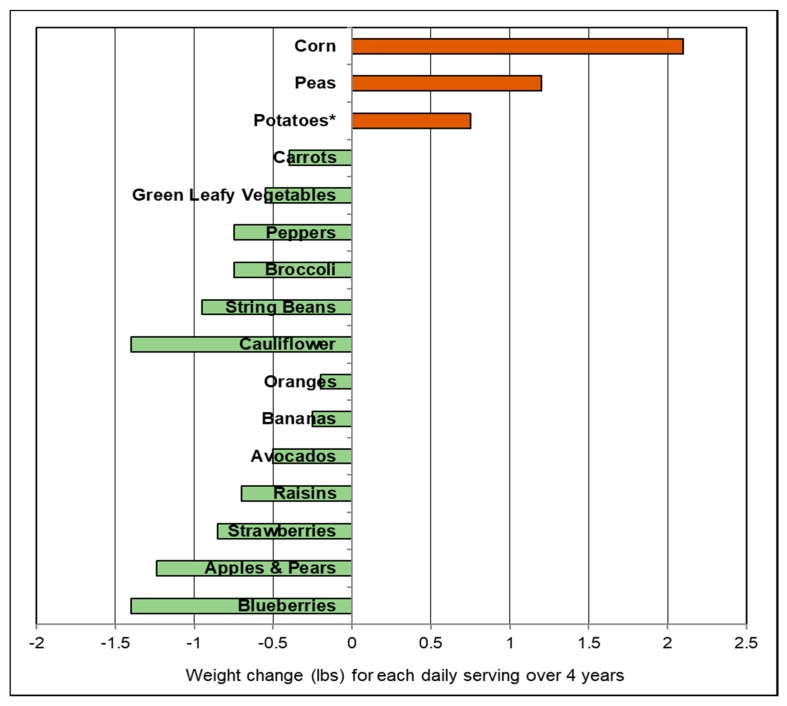
Associations between a daily serving of specific non-juice fruits and vegetables and weight change in US men and women over 4 years from the Nurses’ Health Studies and Health Professionals Follow-up Study (pooled data; multivariate adjusted; *excludes French fries and potato chips) [96].

A number of prospective cohort studies have observed associations between diets containing fruits and vegetables and weight control, weight loss, and risk of obesity. A 2015 systematic review and meta-analysis of prospective cohort studies (17 studies; 563,277 participants) showed that fruit intake was associated with modest reductions in body weight and waist circumference and a reduced risk of obese adiposity by 17% [94]. A 2016 review article concluded that the consumption of increased levels of whole fruit was uniquely protective against weight gain and obesity [95]. Pooled data from 3 US prospective studies including the Nurses’ Health Studies and Health Professionals Follow-up Study (133,468 men and women; baseline normal BMI; 4-year cycles) observed lower weight for intake of total non-juice fruit by 0.53 lb/serving and total vegetables by 0.25 lb /serving [96]. The observed weight change effects for a daily serving of specific fruits and vegetables are summarized in Figure 4. Fiber-rich, very low energy dense blueberries, apples and pears, cauliflower, string beans, peppers and broccoli were among the most effective in the support of weight loss whereas a daily serving of starchy vegetables such as peas, potatoes, and corn were associated with weight gain. The Australian Longitudinal Study on Women’s Health (4287 women; 6 years) observed that women consuming a mean of 117 g fruits and vegetables/day gained 1.1 kg less weight than those consuming a mean of 35 g/day (*p* = 0.002) [97]. The Women’s Health Study (18,146 women; mean 15.9 years) observed that high fruit, but not vegetable intake was inversely associated with the risk of becoming overweight or obese [98]. Overall, this study found no significant associations for fruit and fiber intake with weight gain, whereas higher vegetable intake was associated with greater weight gain. Among women with a body mass index (BMI) <23 kg/m^2^ increased fruit intake was associated with a 13% lower risk of becoming overweight or obese in older age (*p =* 0.01). A 2018 cohort study (4357 middle age participants; 5 years) observed that increased fruit and vegetable intake was associated with a significant decrease in body weight and BMI among Chinese men and similar but not significant change in body weight and BMI among women [99]. A large-scale Canadian population-based survey (26,340 individuals) observed that compared to individuals with <1 fruit serving/day those with >2 fruit servings/day had a 10% lower risk for obesity and 12% lower risk of abdominal obesity (all *p* for trend <0.05) [100]. The National Health and Nutrition Examination Survey (NHANES) observed that increasing the number of servings of whole fruits and non-starchy vegetables relative to total daily food intake was inversely associated with BMI, waist circumference, and fasting insulin [101]. In the Women’s Health Initiative (49,106 postmenopausal, 3 years) each serving of 100% fruit juice/day was associated with a mean 3-year weight gain of 0.4 lbs, partly due to the loss of fiber during processing [102]. For better weight control or modest weight loss overtime, diets containing higher non-juice whole fruits were more effective than fruit juice or vegetables, especially starchy vegetables. 

##### Fiber-Rich Diets

**Figure 5:**
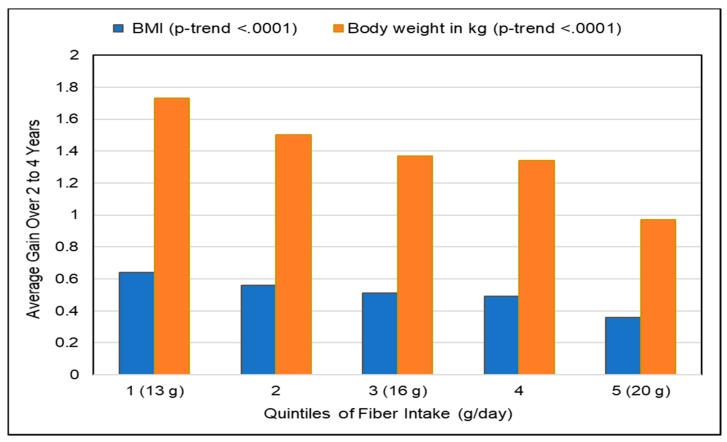
Change in body mass index (BMI) and weight associated with level of fiber intake from The Nurses’ Health Study (74,091 women; age 50 years; 12 years) [104].

Although there are no observational studies which specifically examined associations between higher fruit fiber intake and weight control, waist circumference and visceral fat, there are a number of observational studies for total fiber, which can mirror the expected effects from increased fruit fiber intake, especially from fiber rich fresh fruit because of their low energy density. A systematic review of 14 prospective cohort, case-control and randomized trials found that fiber-rich foods, especially low in energy density, protect against long-term increased weight and waist circumference gain [103]. The Nurses’ Health Study (74,091 US female nurses; 12 years) observed that women with the highest intake of fiber gained an average of 1.5 kg less than those with the lowest intake of fiber (*p*-trend < 0.0001) independent of body weight at baseline and age, and they had a 49% lower risk of major weight gain [104]. In particular, greater increases in fiber intake were associated with less BMI and weight gain every 2 to 4 years (Figure 5). A study in 252 women (20-months) observed that each 1 g/1000 kcal increase in total fiber significantly reduced body weight by 0.25 kg and body fat by 0.25%, by reducing total metabolizable energy intake [105]. Total fiber intake was inversely associated with obesity, metabolic syndrome and elevated CRP risk among US adults in the NHANES 1999–2010 analysis [106]. An EPIC study (48,631 men and women; 5.5 years) observed that an increased level of fiber intake predicted significantly lowered visceral fat, as measured by waist circumference for a given BMI (WC_BMI_), in women [107]. In this study each 10 g increase in fiber intake was associated with a lower WC_BMI_ in women by 0.06 cm and in men by 0.01 cm. The Insulin Resistance and Atherosclerosis Family Study (339 African American adults and 775 Hispanic American adults; 5-years) observed that each 10 g increase in soluble fiber decreased the accumulation of visceral fat tissue by 3.7% (*p* = 0.01) [108]. An intervention trial of Malaysian housewives (255 women; reduced calorie diet to 1200–1500 kcal/day; 6 months) showed that each gram reduction of fiber/1000 kcal intake prevented body fat loss by 243 g/day (*p* = 0.035) [109]. Fruit is an important contributor to help people consume dietary patterns with adequate fiber (≥14 g/1000 kcals; Table 1) which tends to result in leaner phenotypes compared to those consuming low fiber Western diets (≤10 g/1000 kcal) [1,2,11,12,18,19,20,21]. 

#### 3.2.2. Randomized Controlled Trials (RCTs)

##### Fruits and Vegetables

A number of RCTs have evaluated the effects of increased intake of fruits and vegetables on body weight. A 2014 systematic review and meta-analysis (7 RCTs; 1103 subjects; ≥8 weeks) found that total fruits and vegetables including 100% juices in the inclusion criteria show no discernable effects on weight loss [110]. Another 2014 systematic review and meta-analysis excluding 100% juice (8 RCTs; 1026 participants; mean 15 weeks duration) showed that mean increased intake of non-juice total fruits and vegetables by 133 g/day reduced body weight by 0.54 kg (*p* < 0.01), independent of changes in energy intake [111]. The Diabetes Prevention Program multicenter RCT (2624 subjects at risk for diabetes; 1 year) found that weight loss was positively associated with increases in daily servings of non-citrus fruit, and dark green and bright yellow vegetables [112]. A parallel multicenter RCT based on the DASH diet (sub-study of 828 successful weight loss participants) showed that increased intake of total fruits and vegetables was associated with weight loss of 0.3 kg per daily serving during the initial 6-months of the study and continued at a lower rate of 0.04 kg per 6-months for the next 30 months [113]. For apples and pears, a parallel RCT (49 women; isocaloric 3 small apples or pears [0.64 kcal/g] or 3 oat cookies [3.7 kcal/g] added to the usual diet; 10 weeks) showed that apples and pears significantly reduced weight by 0.8–0.9 kg (*p* < 0.001), whereas the cookies increased weight by 0.2 kg (*p* = 0.35) [114]. For avocados, a parallel RCT (51 healthy adults; hypocaloric diet; 12-weeks) found that one Hass avocado [9.2 g fiber]/day or an equally hypocaloric diet without avocados were both equally effective in lowering body weight and BMI by about 3% [115]. Diets containing increased servings of non-juice fruits and vegetables can promote modest weight loss and improved weight loss maintenance.

##### Fiber-Rich Diets

**Figure 6:**
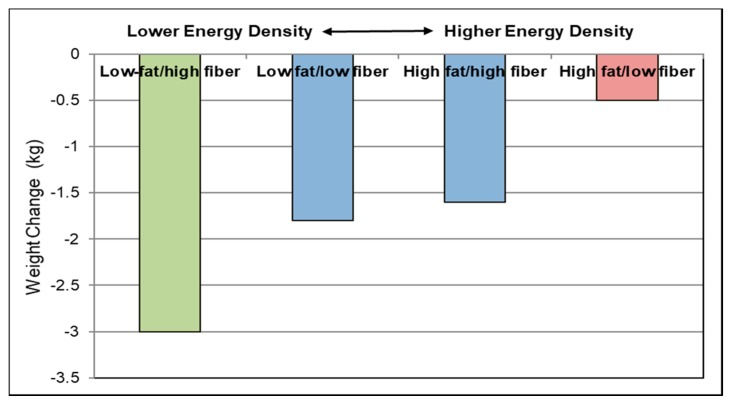
Effect of dietary energy density associated with various levels of fat and fiber intake on body weight in overweight and obese pre-diabetic adults over 3 years in an intensive dietary and exercise program (fiber range 10.9 to 15.6 g/1000 kcal and fat range from <30 to >36.9% energy) [119].

Although there are no RCTs that specifically examined the effects of higher fruit fiber intake and weight control, waist circumference and visceral fat, there are six-high quality RCTs on fiber-rich diets, which are reflective of the expected effects from increased fruit fiber intake on weight control. The Diabetes Prevention Program multicenter RCT reported that an intensive lifestyle intervention group had a 1-year weight loss of 1.45 kg per 5 g fiber intake (*p =* 0.0001), independent of changes in energy intake [112]. A parallel RCT (240 obese metabolic syndrome subjects; 1 year) found that a high fiber diet (goal to consume >30 g fiber/day) was as effective as a reduced energy multicomponent AHA weight loss program after one year [116]. An Australian RCT (72 obese subjects; 12-week duration) showed those consuming 31 g fiber significantly reduced body weight, BMI and % body fat compared to a 20-g fiber/day control [117]. A parallel RCT (113 adults; BMI > 25; randomized into a low-fat vegan diet group at 29 g fiber/day vs. Western habitual diet at 15 g fiber/day; 22 weeks) found that the higher fiber diet group lost significantly more weight by 5.2 kg and waist circumference by 5.5 cm compared to the lower fiber Western diet control group (*p* < 0.0001) [118]. Weight loss of 5% of body weight was more frequently found for subjects in the high fiber group by 49% vs. control group by 11% (*p* < 0.0001). The Finnish Diabetes Prevention Study (522 prediabetic subjects; 4-year duration) showed adequate fiber intake significantly reduced body weight by 2.6 kg (*p*-trend = 0.001) and waist circumference by 1.3 cm (*p*-trend = 0.033) [119]. In this study, the adjusted 3-year weight reduction among those whose diets were both low in fat and high in fiber averaged 2.4 kg more than subjects on a high fat and low fiber diet (Figure 6). A parallel RCT found that centrally obese post-menopausal women who were more dietary adherent to an energy restricted diet lost up to 23% more visceral fat than less adherent subjects, which was correlated with their higher intake of fiber (*p* < 0.001) [120]. These RCTs support that consumption of fiber-rich diets (>28 g fiber/day) can lead to weight loss and improved body composition compared to a low fiber Western diet (≤ approx. 20 g fiber/day).

#### 3.2.3. Mechanisms

##### Colonic Microbiota

A 2018 review article concluded that higher fiber diets tend to be associated with a healthier microbiome (e.g., microflora richness and biodiversity, and higher SCFAs production), which are associated with a lower risk of obesity [121]. Acetate is considered moderately obesogenic, whereas butyrate and propionate are mainly anti-obesogenic. At the macro-colonic microbiota microflora level, individuals with a lean phenotype tend to have a higher *Bacteroidetes* to *Firmicutes* ratio than those with an obese phenotype. A cross-sectional study (68 healthy adults, with a BMI range of 23 to 34 kg/m^2^) observed that lower intake of both fresh whole and dried fruits is associated with higher BMI and had a trend toward reduced levels of the *Bacteroides* group in obese subjects [122]. The combination of fruit fiber such as pectin and polyphenol components can influence the colonic microbiota ecosystem to increase the number of bacterial phyla *Bacteroidetes* and *Actinobacteria*, which are predominant in lean individuals and decrease the prevalence of *Firmicutes* and *Proteobacteria*, which are dominant in obese individuals [98,123]. Several RCTs have evaluated the effects of fiber intake on microbiota health and weight control. A Danish RCT (62 subjects; 66% women; mean age 44 years; *ad libitum* New Nordic diet high in fiber or an average Danish diet lower in fiber; 26 weeks) found an association between the ratio of *Prevotella* (associated with plant-based diets rich in fiber) to *Bacteroides* (P/B) and the effectiveness of fiber-rich diets on weight loss [124]. Subjects consuming a high fiber diet with a high microbiota P/B ratio lost more weight by 2.3 kg compared to subjects with a high fiber diet and a low P/B ratio (*p =* 0.041). The difference in weight loss responsiveness due to P/B ratio may be partially associated with propionate produced by the microbiota. Experimental studies suggest that propionate helps to regulate energy homeostasis by suppressing adipogenesis and inhibiting the excessive formation of adipocytes throughout the body via the inhibition of free fatty acid receptor 2 to block fat accumulation by inhibiting lipid droplet formation and differentiation in the adipose tissue [125]. A crossover RCT (20 non-obese men) suggests that prebiotic fiber stimulated colonic propionate production and appears to have had a role in decreasing the subjective desire for high-energy food leading to reduced energy intake during an *ad libitum* meal by attenuating reward-based eating behavior pathways, independent of changes in plasma PYY and GLP-1 [126]. Also, experimental studies suggest that butyrate may affect energy metabolism indirectly, acting through the gut-brain axis by crossing the blood-brain barrier and activating the vagus nerve and hypothalamus to affect appetite and energy intake [127]. Although more research is needed to better link the various types and sources of fiber with the specific microflora and particular SCFA production that best protects against obesity, a sustained diet high in fiber appears to be associated with a lean phenotype. 

##### Energy Density (ED)

The consumption of low ED foods, especially volumetric foods such as soups or fresh fruits, prior to or as part of a meal can enhance satiety, or reduce hunger and total energy intake during the meal or throughout the day. This is linked to a complex mixture of cognitive, sensory, gastrointestinal, hormonal and neural processes [128,129]. In the natural state most fresh and minimally processed fruits are high in bulk volume and low in ED, ranging from 0.3 to 1.6 kcal/g (Table 1), because of high water (0 kcal/g) and fiber (1–2 kcals/g) content. A recent study showed that humans tend to have a relatively low energy regulatory sensitivity to foods or meals with an ED > 1.75 kcal/g, which can lead to a positive energy balance associated with increased risk of weight gain [130]. For fruits, fresh fruits average 0.6 kcals/g and dried fruits average 2.9 kcal/g as calculated from Table 1, less than many ≥5 kcal/g confectionery, chip or bakery snacks. According to an analysis of the third National Health and Nutrition Survey (NHANES III), the mean ED of the US diet is estimated to be 1.9 kcal/g, which is consistent with the Western diet, low in fruit and vegetable intake and resulting in higher than normal BMIs [131]. A NHANES cross-sectional study from 2005–2008 (9551 adults) observed that higher proportions of energy intake and food weight from low-ED foods like fresh fruits were associated with significantly lower BMI and waist circumference [132]. For example, the substitution of ≥2 servings fresh fruit for higher ED snacks or desserts typical in Western diets can help to reduce total dietary ED to <1.75 kcal/g needed to better support weight control or promote modest weight loss. Providing fresh fruit early in a child’s life is one of the best healthful eating behaviors that is predictive of lower energy intake later in life [133]. A cross-sectional study of weight loss maintainers who lost >10% of their body weight and maintained that loss for ≥5 years reported that they consumed diets with ≤1.4 kcal/g compared to those with weight re-gain who consumed a diet with ≥1.8 kcal/g [134]. The consumption of 2 or more daily servings of fresh fruits as a replacement for higher ED foods such as low fiber, energy rich snacks can help to reduce the total diet energy density level below 1.75 kcal/g to better support weight control and modest weight loss. 

##### Satiety and Energy Intake 

*Fruit Fiber*. Several RCTs have examined the effects of fruit fiber on satiety and energy intake. A systematic review of RCTs on satiety, acute energy intake, total energy intake and body weight found that more viscous or gelling fibers (e.g., pectins, β-glucans and guar gum) reduced appetite more often than less viscous fibers (59% vs. 14%) and lowered acute energy intake (69% vs. 30%) [135]. For pectin, RCTs show that ≥5 g of fruit pectin added to orange juice significantly increased satiety and reduced ice cream intake 4 h later (*p* < 0.001) [136], and 10 g pectin modestly but significantly lowered hunger, increased fullness, and reduced energy intake by 5.6% (*p* = 0.012) compared with the 10 g gelatin and starch control [137]. For orange pomace, a double-blind, crossover RCT found that adding 5.5 g of orange pomace fiber to low fiber orange juice or orange flavor beverage significantly increased satiety vs. a low fiber control juice (*p* < 0.0001) [138]. For banana flour, an RCT showed that adding 8 g unripe banana flour (5 g resistant starch) to soup significantly reduced hunger and increased satiety parameters resulting in a 14% reduction in energy intake compared to the placebo soup [139]. The intake of greater than 5 g of fruit fiber from cell wall components or resistant starch can help to increase satiety, and reduce hunger and energy intake in a subsequent meal. 

**Figure 7:**
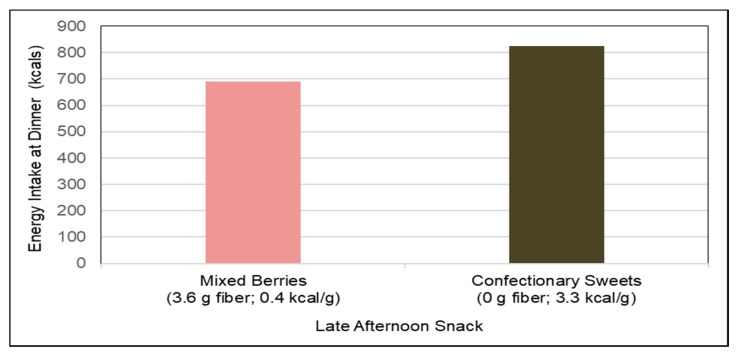
Mean energy intake at *ad-libitum* dinner after a late afternoon 65 kcal pre-dinner snack of mixed berries vs. confectionary sweets (*p* < 0.001) [143].

*Fruit*. A number of RCTs have evaluated the effects of consuming fresh or dried fruits on satiety, hunger and energy intake. Whole fruit consumption results in greater upper intestinal bulk fiber volume, which can delay the absorption of nutrients long enough to deliver a portion of them to the distal ileum. This stimulates the release of a cascade of metabolic *ileal brake* responses associated with the release of satiety hormones (e.g., glucagon-like peptide-1 [GLP-1] and peptide YY), which slow gastric emptying and small bowel transit to reduce acute appetite and energy intake [140,141]. For apples or pears, women adding 3 small apples or pears to the usual diet reduced daily energy intake by 20–25 kcals/day compared to a 1 kcal increase for 3 oatmeal cookies [114] and young adults consuming a whole apple 15 min before a meal significantly lowered energy intake by 15% (*p* < 0.0001) [142] whereas apple sauce reduced energy intake by about 7% and apple juice had no effect. For mixed berries, adults consuming 65 kcal snacks of mixed berries [3.6 g fiber] 60 min before an *ad-libitum* pasta dinner reduced energy intake by 20% more than after an isocaloric sweet confectionery snack (Figure 7) [143]. For fruit snacks, children snacking on grapes and raisins consumed half the energy intake compared to those snacking on chocolate chip cookies and potato chips (about 200 kcals vs. 450 kcals) (*p* < 0.001) [144]. For avocados, overweight adults consuming half an avocado at lunch meals significantly increased meal satisfaction by 23%, decreased the desire to eat by 28% for up-to 5 h [145], and increased GLP-1 blood concentrations during the first 1-h followed by a decline over the next 2-h [146] compared to the avocado-free control lunch. For prunes, adults consuming 5 dried prunes (40 g) as a snack reduced lunch meal energy intake by 100 kcals compared to after an isocaloric low fiber and energy dense snack [147]. Both fresh and dried fruits help to reduce hunger, increase meal satisfaction, and/or decrease energy intake when consumed as snacks or with meals compared to non-fruit control foods.

##### Metabolizable Energy

RCTs show that fiber-rich diets result in higher fecal energy loss, especially unabsorbed fat, compared to low-fiber diets [148,149,150]. A 1978 RCT (6 normal healthy male subjects; isocaloric diets; 5 to 8 days) found that fecal macronutrient energy loss on a low-fiber diet was less than half of that observed on a high-fiber diet [151]. The consumption of >25 g fiber/day can lead to the excretion of 3–4% of macronutrient energy in the feces, which is equivalent to 80 kcals in a 2000-kcal diet. An energy balance intervention (8 healthy female students) showed that subjects with high fiber diets rich in fruits and vegetables had a significantly higher fecal metabolizable energy content of 220 kcal/day compared to 108 kcal/day for subjects with low fiber diets [152]. A small RCT (4 lean and 4 obese adults) found that obese subjects had lower mean fecal macronutrient energy loss (or more efficient energy absorption) than lean subjects [153]. 

### 3.3. Cardiovascular Disease (CVD)

Cardiovascular diseases are the leading cause of morbidity and mortality worldwide and healthy dietary patterns including fiber rich fruits, vegetables, whole-grains and nuts have an important role in prevention [154,155]. 

#### 3.3.1. Fruit and Fruit Fiber

##### Vascular Aging (Atherosclerosis)

**Figure 8:**
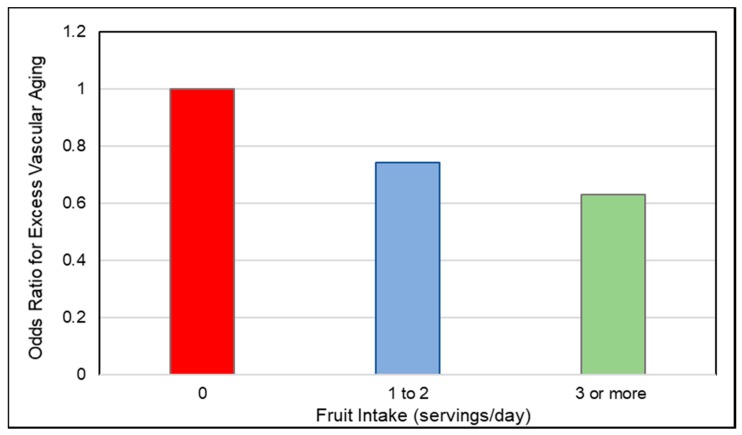
Association between the number of fruit servings/day and excessive vascular age from pooled estimates of World Health Organization (WHO) and US National Health and Nutrition Examination Survey (NHANES) data among adults aged 30–74 years (*p =* 0.038) [156].

**Figure 9:**
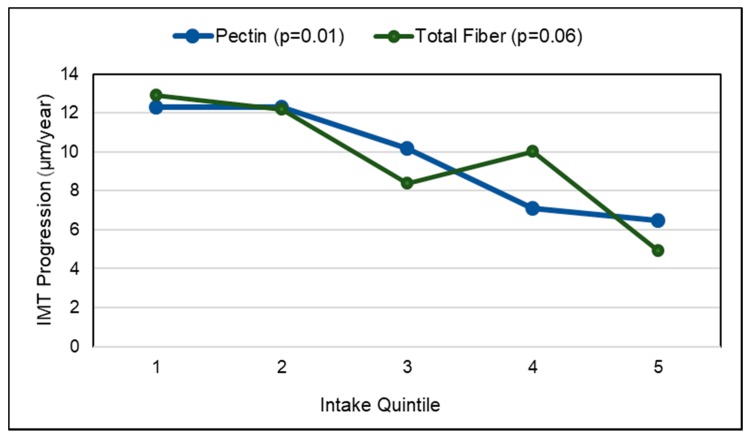
Progression of intima-media thickness (IMT) in the common carotid-artery by quintile of fiber intake among 500 participants in the Los Angeles Atherosclerosis Study [157].

*Observational Studies*. Vascular aging with the build-up of atheromatous plaques in medium-and large-sized blood vessels, raises CVD risk, which can be assessed by measuring the common carotid intima-media thickness (IMT), carotid artery stiffness or abdominal aortic calcification [154,155,156]. A 2017 analysis of data from cardiovascular disease-free adults in the World Health Organization (WHO) Study on Global Aging and Adult Health (29,094 adults) and US NHANES (6726 adults) observed that people with higher fruit intake had lower odds of excessive vascular aging as calculated by the Framingham CVD risk equation (Figure 8) [156]. The Los Angeles Atherosclerosis Study (573 participants; mean age 50 years; 2-year follow-up) observed that a higher intake of pectin significantly slowed IMT progression (Figure 9), which appears to be mediated by lowering the ratio of total to HDL cholesterol [157]. The Cardiovascular Risk in Young Finns cohort study (1809 participants; 27 years) observed that carotid IMT progression was inversely associated with fruit intake in childhood [158]. In a Mediterranean cohort of young adults, the Seguimiento Universidad de Navarra Follow-up study (17,000 participants; 10.3 years) observed an inverse association between fruit consumption and fruit fiber and CVD risk with a risk reduction of approximately 50% each (*p*-trend = 0.024) [159]. The consumption of ≥two daily servings of fruit (160 g/day) reduced CVD risk by 38% in young Mediterranean populations (*p =* 0.045). An Australian cohort study (1510 elderly women; 5 years) observed that each 50 g/day apple consumption (approx. ½ of a small apple) was significantly associated with 24% lower odds of having severe abdominal aortic calcification (*p =* 0.009) [160]. A Chinese prospective study (70,047 adults with physician-diagnosed CVD) observed a 29% reduction in CVD mortality for each daily 100 g intake of whole fruit [161]. The UK Women’s Cohort Study (30,458 women; 17 years) observed that each serving of fresh fruit or dried fruit/day significantly reduced risk of mortality from CHD by 11% and CVD by 8% (*p =* 0.007) [162]. A 2013 systematic review and meta-analysis found a lowered risk of CHD or CVD by 8% per each 4 g intake of fruit fiber/day, with substantial heterogeneity between type of fruit [163]. Although more research is needed, the sustained intake of whole fruits and fruit fiber has been associated with a reduced rate of vascular aging and lower risk of mortality from CVD. 

*Intervention Trial*. An acute intervention trial (31 newly diagnosed CVD patients; elevated CVD risk profile; high fiber diet primarily consisted of fruits, vegetables, avocados and seeds) found that this diet significantly improved blood lipids and other CVD risk factors to reduce vascular aging compared to baseline values and rapidly reduce CVD risk profiles (Table 2) [164]. In newly diagnosed CVD patients, the consumption of an acute healthy diet rich in fruits and vegetables with approximately 50 g fiber/day has the potential to realign major CVD risk factors back to normal levels before a healthy dietary pattern is adapted for a longer-term CVD risk management. 

##### Blood Lipids

**Figure 10:**
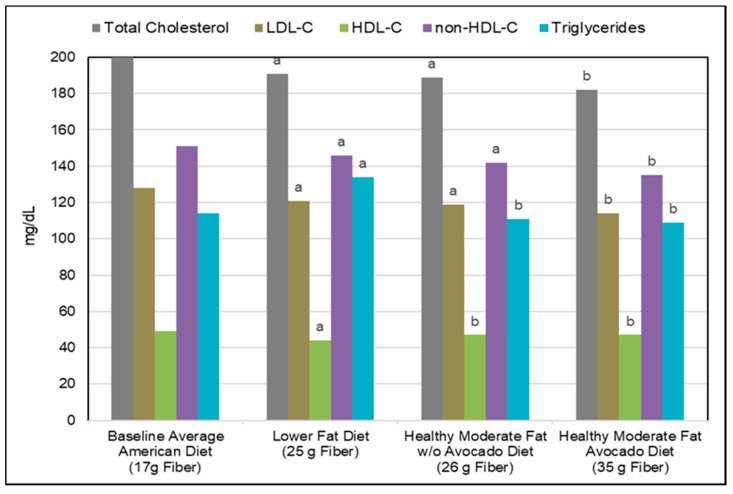
Effect of fiber and fat on total and LDL-cholesterol in a crossover Randomized Controlled Trial (RCT) of 45 overweight or obese adults over 5 weeks (based on 2100 kcal/diets; total fat 34% of energy for all diets except 24% for the low-fat diet; protein approx. 16% of energy for all diets; saturated fat energy 13% for the American diet, 7% for the low-fat diet and 6% for the two moderate fat diets with healthy vegetable oil vs. 1 avocado. Values with different superscript letters (a and b) are significantly different (*p* < 0.05) [169].

*RCTs*. A number of RCTs and meta-analyses of RCTs show that the consumption of pectin and whole fruits can lower total and LDL-cholesterol (LDL-C) levels and other cardiometabolic risk factors. The primary mechanism is related to the increased fruit fiber bulking, especially from viscosity/gelling of pectin, in the ileal region of the small intestine reducing the efficiency of bile acid reabsorption (the enterohepatic circulation) and leading to increased uptake of circulatory LDL-C by the liver to compensate for reduced availability of bile acids [165]. For citrus and apple pectin, each gram of pectin added to the diet has a mean lowering effect on serum total cholesterol by 1.7 mg/dL/g and LDL-C by 2.2 mg/dL/g [166]. A crossover RCT (30 mildly hypercholesterolemic subjects; age 18–70 years; 6 g/day of different types of pectins; 3 to 4 weeks; 1-week washout) demonstrated that the most effective sources are citrus and apple pectin DE-70, which reduced LDL-C by 6–7% [167]. Also, a crossover RCT showed that apple products lowered total and LDL-C levels in direct relation to the apple pectin content [168]. A whole apple (2.9 g pectin) was approximately 3 times more effective in lowering LDL-C than apple juice (0.5 g pectin). For avocados: (1) A crossover RCT found that eating a daily avocado (9.2 g fiber and estimated 3.6 g pectin) as part of a moderate 34% energy from healthy fat diet significantly reduced total-cholesterol by 6.5 mg/dL, LDL-C by 5.3 mg/dL and non-HDL-C by 6.2 mg/dL compared to a similar energy matched moderate healthy fat diet without an avocado (Figure 10) [169]. (2) A crossover RCT (31 middle age adults; breakfast meal with a half or whole avocado vs. a higher carbohydrate control meal without avocado; 6 hrs postprandial) demonstrated both a half or whole avocado significantly improved postprandial glycemia, insulinemia and flow mediated vasodilation (FMD) compared to a control breakfast [170]. The consumption of a whole avocado significantly lowered levels of triglycerides (TG) and promoted high levels of larger HDL particles. (3) A 2016 meta-analysis of RCTs with all primary blood lipid outcomes (8 crossover RCTs and 2 parallel RCTs; 229 subjects) found that diet substitutions of avocado for other dietary fats significantly decreased total cholesterol by 19 mg/dL, LDL-C by 17 mg/dL, and TG levels by 27 mg/dL [171]. HDL-C was insignificantly decreased by 0.18 mg/dL. (4) A 2018 meta-analysis (7 RCTs; 220 subjects) showed that consuming an avocado insignificantly reduced total cholesterol by 3.4 mg/dL, LDL-C by 3.5 mg/dL and triglycerides by 12 mg/dL, and significantly increased HDL-C by 2.8 mg/dL compared to a diet without an avocado [172]. For prunes, a crossover RCT (41 hypercholesteremic men; 12 prunes (100 g/6 g fiber) vs. grape juice control; 8 weeks) showed that prunes significantly lowered LDL-cholesterol by 7.7 mg/dL compared to the grape juice control (*p* = 0.02) [173]. Prunes also increased excretion of the bile acid lithocholic acid by 25 mg/g dry stool. For berries, a meta-analysis of berry consumption (22 RCTs; 1251 subjects; variety of whole berries, juices, and smoothies) concluded that whole berries and smoothies significantly reduced mean LDL-cholesterol by 8.1 mg/dL (*p* = 0.003), systolic blood pressure by 2.7 mmHg (*p* = 0.04), BMI by 0.36 kg/m^2^ (*p* < 0.00001), hemoglobin A1c (HbA1c) by 0.20% (*p* = 0.04) and tumor necrosis factor-α (TNF-α) by 0.99 ρg/mL (*p* = 0.04) compared to control diets [174]. Whole fruit fiber, especially pectin from citrus, apples, avocados, prunes, and berries, has an active role in helping to reduce LDL- and non-HDL-cholesterol by lowering bile acid reabsorption to attenuate CHD and CVD risk. 

##### Hypertension

**Figure 11:**
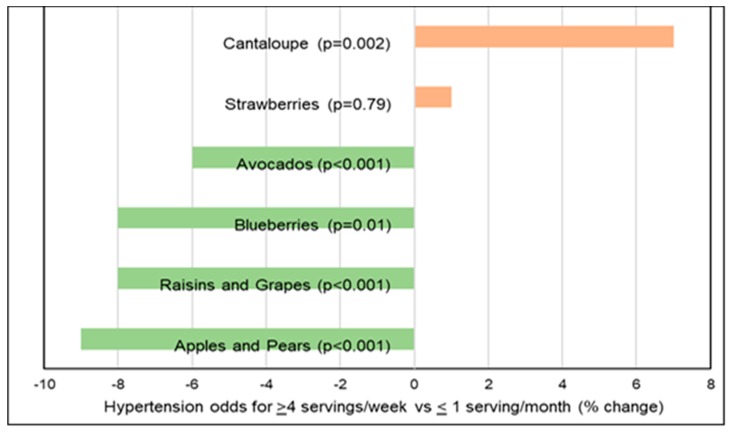
Change in the risk of hypertension associated with fruit consumption from pooled Nurses’ Health Study, Nurses’ Health Study II, and Health Professional Follow-up Study data [175].

**Figure 12:**
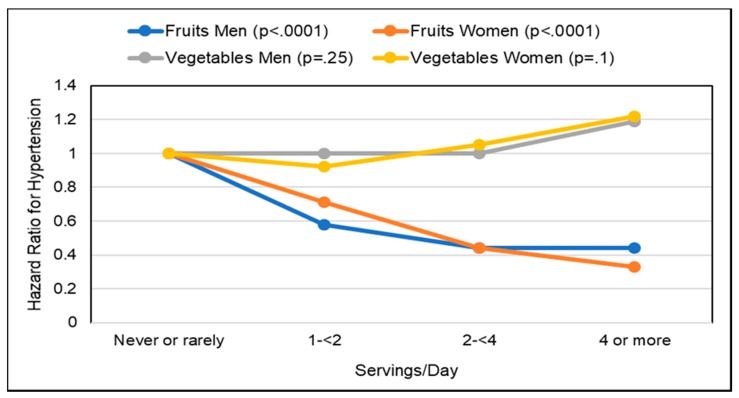
Association between the number of fruit or vegetable servings and hypertension risk in a cohort of middle-aged and older Koreans [182].

*Observational Studies*. A number of prospective cohort and cross-sectional studies have observed associations between the effects of increased fruits and vegetables on blood pressure (BP) and the risk of hypertension. An analysis of 3 large, long-term cohorts (187,453 participants from Nurses’ Health Study, Nurses’ Health Study II, and Health Professionals Follow-up Study; >20 years) observed a lower mean risk of hypertension for whole fruit by 8% compared to a 5% lowering for vegetables (≥4 servings/day vs. ≤4 servings/week) [175]. The effect of specific fruits on risk of hypertension is summarized in Figure 11. A meta-analysis (28 cohort studies) observed an inverse association for the risk of hypertension for 30 g whole grains/d by 8%, 100 g fruits/d by 3% and 200 g dairy/d by 5%, whereas there was a positive association for 100 g red meat/d by 14%, 50 g processed meat/d by 12%, and 250 mL sugar sweetened beverages/d by 7% [176]. Two meta-analyses: (1) 25 studies; 334,468 participants [177] and (2) 7 cohort studies; 185,676 participants [178] observed that each daily increased fruit serving intake lowered hypertension risk by approximately 2–3% per 80-g serving. The Women’s Health Study (28,082 health professionals; 13 years) observed that women who had a higher intake of fruits but not vegetables had significantly reduced hypertension risk, after adjustment for lifestyle and dietary factors [179]. A cross-sectional study (806 men; age 40–69 years) observed that fruit fiber was inversely associated with BP (*p* = 0.07) with no significant association observed for cereal or vegetable fiber [180]. An increase in one daily portion of fruit to the diet was associated with a reduction of approximately 0.7 mm Hg in systolic BP and 0.5 mm Hg in diastolic BP after adjusting for calcium, potassium, magnesium, education, and physical activity. The Spanish Seguimiento University of Navarra (SUN) study (8594 participants; 6 years) observed a significant inverse association between fruit intake and BP, but not for vegetable intake [181]. The Korean Genome and Epidemiology Study (4257 adults without hypertension at baseline; 8 years) observed that a higher intake of fruit was associated with a lower risk of hypertension in middle-aged and older Koreans (Figure 12] [182]. A US cross-sectional study (163 adolescents) observed that fruit intake was inversely associated with BMI and diastolic BP [183]. The INTERMAP population study (4680 adults from Japan, China, the United Kingdom, and the US) observed a significant reduction in systolic BP by 1.7 mm Hg for each 7-g fiber/1000 kcals increase, independent of potassium and magnesium [184]. The Health Professionals Follow-up Study (30,681 men; 40–75 years; 4 years) observed that men consuming <12 g fiber/day had a 57% increased risk of hypertension compared to men with an intake of >24 g/day and fruit fiber but not vegetable or cereal fiber was inversely associated with hypertension risk [185]. The Nurses’ Health Study (41,541 US women; 4 years) observed that women consuming <10 g fiber/day had a 109% greater risk of elevated BP compared to women consuming ≥25 g fiber/day [186]. A NHANES analysis of data between 2007–2014 (18,433 US adults; ≥18 years) showed that the intakes of cereal and vegetable fibers were associated with significantly lower hypertension risk by 18–20%, but fruit fiber was insignificantly associated with a 14% lower risk of hypertension, which is inconsistent with previous studies and potentially due to a younger demographic and the methodology used [187]. The consumption of whole fruits and fruit fiber lowers the risk of hypertension and reduces BP more effectively than vegetables, especially in middle aged or older adults.

*RCTs*. A number of RCTs and systematic reviews or meta-analyses of RCTs have evaluated the effects of increased fruit and vegetable intake on BP. A Cochrane systematic review (2 RCTs; 891 generally healthy adults; 6–12 months) found that the consumption of at least 5 servings of fruits and vegetables significantly reduced mean systolic BP by 3.0 mm Hg compared to low fruit and vegetable intake [188]. An RCT (459 hypertensive and normotensive adults) showed that hypertensive subjects significantly reduced systolic BP by 7.2 mm Hg (*p* < 0.001) and diastolic BP by 2.8 mm Hg (*p* = 0.01) with 8–10 servings/d vs. approximately 4 servings/d of fruits and vegetables [189]. Non-hypertensives had an insignificant reduction in systolic BP by 0.8 mm Hg and diastolic BP by 0.3 mm Hg. A dose response RCT (117 adults; 12 weeks) showed that each serving of fruits and vegetables significantly increased forearm blood flow by 6% in hypertensive subjects [190]. An RCT (690 healthy middle age adults; mean BP 130/79 mm Hg; 6 months) found that at least 5 fruit and vegetable servings/day significantly reduced systolic BP by 4.0 mm Hg and diastolic BP by 1.5 mm Hg compared to 3.5 fruit and vegetable servings/day [191]. A double-blind RCT (52 middle aged women; 8 weeks) showed that the Dietary Guidelines for Americans with adequate fruit and vegetable intake significantly lowered systolic BP by 9 mm Hg without weight loss compared to the typical American diet [192]. A systematic review and meta-analysis (20 RCTs; 1917 adults) found that the DASH diet (8–10 fruits and vegetables servings/d) significantly decreased systolic BP by 5.2 mm Hg and diastolic BP by 2.6 mm Hg (all *p* < 0.001) with greater reductions in subjects with higher baseline BP and/or BMI [193]. A 2018 meta-analysis (22 RCTs; 1430 participants; 7 weeks) found that viscous soluble fibers including fruit pectin have a small but significant mean lowering effect on systolic BP by 1.6 mm Hg and diastolic BP by 0.4 mm Hg compared to control diets [194]. A 2016 Cochrane Systemic review (8 RCTs; 661 adults with high CVD risk; >12 weeks) found that increased fiber intake significantly lowered mean systolic BP by 1.9 mm Hg and diastolic BP by 1.8 mm Hg [195]. Two 2005 meta-analyses of RCTs showed that an increase in fiber intake by 11 g/day can significantly lower mean systolic BP by 6 mm Hg and mean diastolic BP by 4.2 mm Hg in hypertensive subjects [196,197]. RCTs generally show that increased fruit and vegetable intake results in a significant reduction of BP in older, overweight, prehypertensive or hypertensive subjects compared to younger, normal weight and normotensive subjects.

*Mechanisms.* There are a number of whole fruit and fruit fiber related mechanisms associated with BP and hypertension lowering effects: (1) reducing risk of weight gain or obesity; (2) lowering risk of insulin resistance, an important pathophysiological influence on the development of endothelial dysfunction; (3) promoting healthier lipoprotein profiles to slow the rate of vascular aging (arterial plaque build-up); (4) attenuating elevated systemic inflammation and LDL-oxidation risk; and (5) stimulating a healthier colonic microbiota and increased fermentation to SCFAs to upregulate receptor expression in the kidneys or other blood vessels thought to be associated with better BP regulation [198,199,200,201].

### 3.4. Type 2 Diabetes (Diabetes)

Prevention of diabetes is determined by controlling blood glucose and insulin, and systematic inflammation to preserve insulin receptor and pancreatic β cell function, which is achievable by the implementation of early and sustainable lifestyle measures including modifying dietary and physical activity habits which induce better weight control or modest weight loss, a preferred approach to drug therapy, when possible, due to its safety, efficacy, and cost [202,203,204].

#### 3.4.1. Fruit and Fruit Fiber

##### Observational Studies

**Figure 13:**
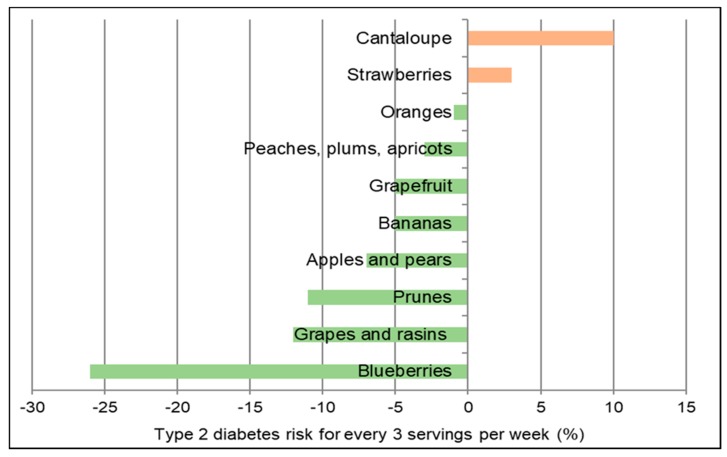
Effect of 3 servings per week of fruit varieties on type 2 diabetes (diabetes) risk from pooled data from US men and women in the Nurses’ Health Studies and the Health Professionals Follow-up Study [208].

**Figure 14:**
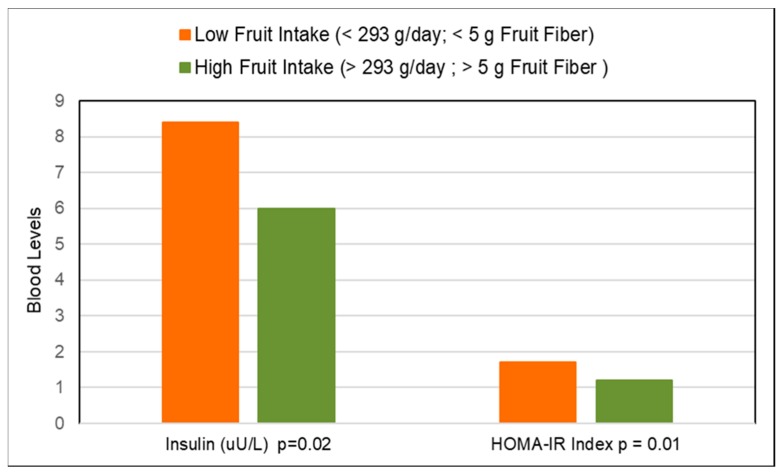
Association between fruit and fruit fiber intake on insulin sensitivity measures in 40 normal-weight young women with low to normal TNF-α values [214].

Low glycemic index (GI) foods such as fiber containing whole fresh fruits can be healthy substitutes for high GI foods, as part of any meal, snack or dessert to reduce the risk of and management of diabetes. A 2016 meta-analysis (17 cohort studies) found that total fruit reduced diabetes risk (highest to lowest intake) by 9% and blueberry intake significantly reduced risk by 25% [205]. A 2015 meta-analysis of fruit (9 cohort studies; 403,259 participants) found a non-linear association for fruit intake and diabetes risk (*p* for nonlinearity <0.001) with a threshold of 200 g/day total fruit intake reducing risk of diabetes by 13% [206]. A Finnish prospective study observed that individuals in the highest quartile for intake of fruits, berries, and vegetables (with the exclusion of potatoes and fruit juices) had a significant 24% reduction in diabetes risk compared to those in the lowest quartile [207]. A pooled analysis of the Nurses’ Health Studies and the Health Professionals Follow-up Study observed a high degree of heterogeneity in the effect of whole fruits on diabetes risk per 3 weekly servings (Figure 13) [208]. The 2017 China Kadoorie Biobank cohort study (0.5 million adults; 7 years) observed that among those without diabetes at baseline, higher fruit intake was associated with significantly lower risk of developing diabetes by 12% compared to non-consumers (*p* < 0.001) [209]. Among those individuals who had diabetes prior to the start of the study, consuming fresh fruit more than 3 days/week was associated with a 13% to 28% lower risk of developing diabetes-related complications affecting large blood vessels (e.g., ischaemic heart disease and stroke) and small blood vessels (i.e., kidney diseases, eye diseases, and neuropathy) compared to individuals who consumed fruit ≤one day/week (*p* < 0.001). A meta-analysis (9 cohort studies; 341,668 participants) observed that high versus low fruit fiber intake lowered diabetes risk by 6% but cereal fiber (11 cohort studies; 389,047 participants) reduced diabetes risk by 23% [210]. The EPIC-Potsdam Study (9702 men and 15,365 women; 7 years follow-up) observed that increased soluble fiber was twice as effective in reducing diabetes risk as insoluble fiber (17% vs. 7%) [211]. The Australian Longitudinal Study (3607 young women; 12 years) observed that women in the highest quartile of fiber intake had a 33% lower risk of developing gestational diabetes (*p =* 0.05) [212]. Higher fruit intake, especially fiber rich and low GI varieties, reduced gestational diabetes risk by 5% per 50 g/day whereas cereal intake may increase gestational diabetes risk by 5% per 20 g/day, especially high GI and low fiber sources. The EPIC-Norfolk Study (5996 healthy adults) observed that participants who reported consuming fruit ≥5 times/week (20 g fiber/day) vs. never or seldom consuming fruit (13 g fiber/day) had significantly lower mean adjusted HbA1c by 0.1% (*p* = 0.007) [213]. A cross-sectional study (40 women) observed that higher intake of fruits (>293 g/day; especially apples and citrus fruits) was related to significantly lower fasting blood insulin and HOMA-IR (Figure 14) , which was mediated by higher fiber and antioxidant intake [214]. The Japan Diabetes Complications Study (978 diabetes patients; HbA1c ≥6.5%; 8-years) observed that the consumption of 253 g fruit/day reduced the incidence of diabetic retinopathy by 52% compared to intake of 23 g fruit/day (*p* < 0.01) [215]. A cross-sectional study (264 women) observed that each 1 g increase in soluble fiber decreased HOMA-IR by 0.11 (*p =* 0.006) [216]. Also, women with high soluble fiber intake had a 48% lower risk of insulin resistance vs. women with low soluble fiber intake. A cross-sectional analysis of the Nurses’ Health Study observed that fruit fiber intake was inversely associated with HbA1c concentrations (*p*-trend < 0.03) [217]. A Japanese longitudinal study (190 adults 5 months) observed that increasing the diet’s fiber to total carbohydrate ratio was associated with significant reductions in HbA1c (*p* < 0.001) [218]. The Cohort Diabetes and Atherosclerosis Maastricht prospective study (303 adults; 7 years) observed that combined increased intake of fiber and monounsaturated fatty acids (MUFA) were the primary nutrients associated with better pancreatic β-cell function [219]. A cross-sectional study (68 adults) observed that lower intake of whole fruit was associated with increased diabetes risk due to higher free radical and pro-inflammatory status [122]. The Nurses’ Health Study (70,025 women; mean baseline age 50 years; 24 years follow-up) observed that both total and fruit fiber were associated with 20% lower odds of diabetes compared to a 30% lower risk for cereal fiber [220]. Also, a higher starch or total carbohydrate to total fiber ratio was associated with an approximately 10% increased odds of diabetes risk. Observational studies generally observe that higher fiber containing fruits are associated with lower diabetes risk and better control of diabetes risk biomarkers such as HOMA-IR, HbA1c and fasting insulin, and lower risk of diabetic retinopathy.

##### RCTs

**Figure 15:**
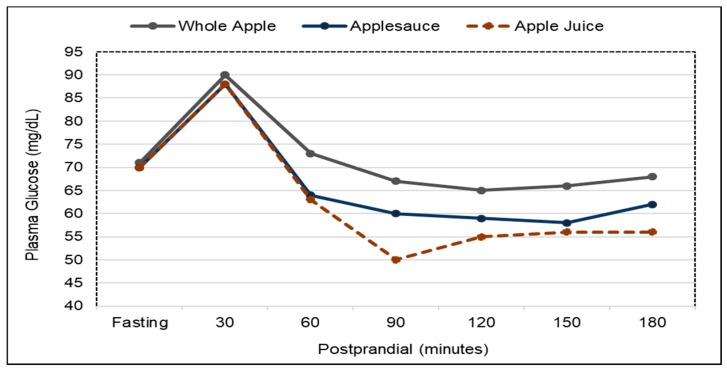
Mean postprandial plasma glucose levels after eating whole apples, applesauce, and apple juice (applesauce and apple juice plasma glucose significantly fell below whole apple levels after 75 to 180 min [*p* < 0.05]) [221].

**Figure 16:**
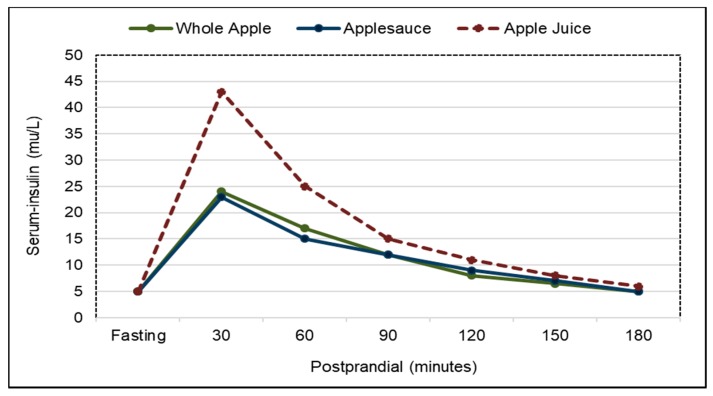
Mean postprandial serum insulin levels after eating whole apples, applesauce, and apple juice with serum insulin significantly increased for apple juice above whole apples and applesauce after 10 to 40 min (*p* < 0.01]) [221].

*Postprandial Glycemic Control*. Eating whole fruit increases gastrointestinal bulk from chewed fruit pulp and edible skins and viscosity from soluble fiber to the stomach which delays its emptying compared to purees or juice, and attenuates postprandial sugar absorption from the small intestine and insulin secretion to prevent the potential postprandial dip in blood glucose and increase in insulin often observed after consuming fruit juices [221]. For apples, a crossover RCT in healthy adults found that as the degree of apple processing increases and the fruit is broken down into smaller particles and cell walls further disrupted, the rate and completion of digestion in the small intestine significantly increases, which results in higher postprandial plasma glucose and serum insulin levels (Figure 15 and Figure 16) [221]. In another similar RCT, the glycemic and insulinemic effects of whole oranges and orange juice responded similarly to whole and processed apples [222]. For orange pomace, a double blind, crossover RCT showed that orange pomace, a fiber rich orange juice processing by-product, added to a high carbohydrate breakfast beverage significantly lowered glycemic response by 36% and insulinemic response by 23% in overweight men compared to the control beverage [223]. For a berry puree, a crossover RCT found that adding a berry puree to bread during breakfast significantly reduced postprandial glycemic and insulin responses by 38% compared to plain bread [224]. For a mixture of whole fruits, a crossover RCT found that a high carbohydrate breakfast high in whole fruit significantly reduced glucose response by 18% compared to a breakfast low in whole fruits [225]. For avocados, a single-blind crossover RCT (31 middle age, overweight adults; 6 h) showed that consuming test meals containing either a half or full avocado significantly decreased the postprandial glycemic and insulinemic responses to meals over 6 h compared to control meals (both *p* < 0.0001) [170]. Postprandial glucose peak concentrations were also significantly lower after consuming both avocado-containing meals at approx. 7 mmol/L compared to the control meal at approx. 8.1 mmol/L (*p* < 0.0001). Similar effects were observed in postprandial insulin peak concentrations after avocado-containing meals compared to a control meal (*p* < 0.005). There were no significant differences between the half and whole avocado meals on glucose and insulin response. Also, another crossover RCT showed that consuming half an avocado with a lunch meal significantly attenuated postprandial blood insulin by up to 37% (*p* = 0.04) within 30 min and lowered blood insulin levels over a 3 h postprandial period compared to the similar avocado-free control lunch meal [145]. For dried bananas, a crossover RCT showed that adding dried whole banana powder (rich in resistant starch) to a milkshake reduced postprandial glucose response by 43% (*p* = 0.03) compared to the control milkshake [226]. For raisins, a crossover RCT found that adding raisins to a meal resulted in significantly reduced postprandial glucose and insulin responses when compared to a raisin-free meal (*p* < 0.05) [227]. The bulking and soluble, viscous fibers from both fresh and dried fruits help to attenuate postprandial insulin responses to protect against the risk of insulin resistance and β-cells dysfunction and senescence, major factors in developing diabetes [228,229,230].

*Fasting Biomarkers*. A number of RCTs show that whole fruit, especially fruits with higher fiber and polyphenols, improved biomarkers of diabetes risk. For berries: (1) A meta-analysis (22 RCTs; 1251 subjects) observed that mixed berries significantly reduced mean fasting glucose by 1.8 md/dL, hemoglobin A1c (HbA1c) by 0.2%, BMI by 0.4 kg/m^2^, and tumor necrosis factor-α (TNF-µ) by 1.9 pg/mL compared to non-fruit controls [174]; and (2) A double-blinded, placebo-controlled RCT (32 obese, nondiabetic, and insulin-resistant subjects; 6 weeks) showed that whole blueberry smoothies significantly improved insulin sensitivity by 1.7 mg × kg FFM 21 × min21 compared to the placebo smoothie (0.4 mg × kg FFM 21 × min21) (*p* = 0.04) [231]. For raisins, a parallel RCT (46 adults; raisins vs. conventional processed snacks; 12 weeks) showed that raisins significantly lowered HbA1c by 0.12% compared to commonly consumed carbohydrate-rich processed snacks (*p = 0*.036) [232]. For high vs. low fruit, a parallel RCT (63 men and women with newly diagnosed type 2 diabetes; fruit intake 319 vs. 135 g/day; 12 weeks) found that the higher fruit group directionally reduced mean HbA1c by 0.2%, lowered body weight by 0.8 kg and waist circumference by 1.3 cm compared to the lower fruit group [233]. Two meta-analyses of RCTs in patients with diabetes estimated that fiber-rich diets increased by a mean of 18 g fiber/day and/or by 3–15 g of viscous soluble fiber supplementation, such as fruit pectin, can have significant beneficial effects on glycemic control by reducing mean fasting blood glucose levels by 10–15 mg/dL and HbA1c by 0.26–0.55 mg/dL compared to a control over 3–24 weeks [234,235]. The protective mechanisms associated with whole fruits and lower diabetes risk include improved weight control, satiety, glucose tolerance, insulin sensitivity, microbiota ecosystem health and reduced systemic inflammation [236,237,238]. In recent years, fiber’s role in promoting and maintaining colonic microbiota health has been shown to have a critical effect on alleviating risk of and in management of diabetes. A 2018 RCT found that when fiber dependent-SCFA producing bacteria were present with greater diversity and abundance in the microbiota, the subjects had lower HbA1c levels, partly via increased glucagon-like peptide-1 production [239]. Increased fiber intake, beneficial microflora and SCFAs are protective against T-cell infiltration into pancreatic β-cells. A double-blind crossover trial (16 healthy adults) showed that soluble fiber fermentation by microbiota resulting in the SCFA propionic acid, increased hepatic derived heptadecanoic acid (17:0), which is inversely associated with diabetes risk [240]. Both fresh and dried fruits help to maintain healthy glycemic and insulinemic risk biomarker levels, which appears to be in response to fruit fiber effects on promoting a healthy microbiota ecosystem, to protect against diabetes risk or associated co-morbidities.

### 3.5. Metabolic Syndrome

Metabolic syndrome is defined as a pathologic condition characterized by abdominal obesity, insulin resistance, hypertension, and hyperlipidemia by the WHO [241]. The two basic risk factors are the increase in consumption of high calorie-low fiber foods and the decrease in physical activity due to motorized transportations and sedentary leisure time activities. The syndrome is linked to the increased prevalence of diseases like diabetes, CVD, and other disabilities, which are associated with higher cost of health care and loss of potential economic activity in the trillions of dollars, worldwide.

#### 3.5.1. Fruit

Observational studies support the beneficial effects of adequate whole fruit intake in helping to lower the risk of metabolic syndrome. Two 2018 meta-analyses of observational studies found that the higher intake of whole fruit was associated with a lower risk of metabolic syndrome by 15–19% [242] compared to an 11% risk reduction for vegetables [243]. A Korean cross-sectional study (243 healthy non-diabetic participants) observed that higher fruit intake was associated with increased HDL-cholesterol, and lower waist circumference, triglycerides, LDL-cholesterol, and insulin resistance, which are important attributes associated with reducing the risk of metabolic syndrome [244]. Two intervention studies show that whole berries are among the most effective fruit types in lowering hypertension, insulin resistance, dyslipidemia, and lipid oxidation related to metabolic syndrome risk [245,246]. A NHANES analysis (2001–2008) observed that avocado consumers had significantly smaller waist circumference, higher HDL-C and 50% lower risk for metabolic syndrome compared to non-consumers [247]. A comprehensive systematic review concluded that avocados provide strong protection against metabolic syndrome risk and help mitigate metabolic syndrome symptoms [248]. A NHANES analysis (2001–2011) observed that raisin consumers compared to non-consumers had significantly lower waist circumferences and a 54% reduced risk of metabolic syndrome [249]. A cross-sectional study (486 Tehrani female teachers) observed that women in the highest quintile of fruit intake had a 34% lower risk of metabolic syndrome and 25% reduction in CRP levels compared to women eating the lowest fruit intake [250]. Healthy diets rich in whole fruits help to attenuate metabolic syndrome risk because of their low energy density, high fiber to total carbohydrate ratios, and high content of antioxidant and anti-inflammatory vitamins (C and E) and phytochemicals (polyphenols and carotenoids).

#### 3.5.2. Fruit Fiber

**Figure 17:**
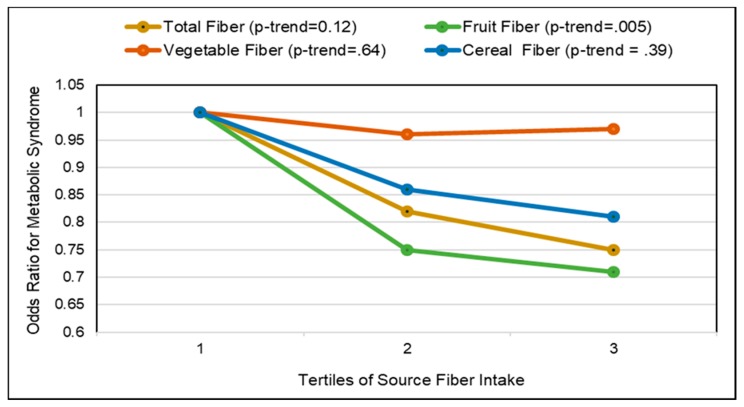
Odds ratio for Metabolic Syndrome by categories of fiber sources of intake (median) at baseline examination from the Tehran Lipid and Glucose Study (multivariate adjusted; total fiber (g) = 10.2, 14.1, and 21.6; fruit fiber (g) = 1.2, 2.7 and 5.2; vegetable fiber (g) = 1.2, 2.6 and 5.9; and cereal fiber (g) = 1.6, 3.2 and 4.5) [252].

Adequate fiber intake is associated with reduced odds of having metabolic syndrome. A 2017 meta-analysis (8 cross-sectional and 3 cohort studies; 28,241 participants) observed a curvilinear relationship between fiber consumption and prevalence of metabolic syndrome [251]. Fiber intake levels including fruit fiber were inversely associated with metabolic syndrome with 30–40 g fiber/day lowering risk by 27%. The Tehran Lipid and Glucose Study (1582 adults; 3-years) observed that fruit fiber provided the best protection against metabolic syndrome compared to other fiber sources (Figure 17) [252]. The risk of metabolic syndrome was reduced by 6% for each 1 g of fruit fiber intake per 1000 kcals. A 2018 Mexican longitudinal study (30 participants with metabolic syndrome; increased daily fiber intake by 15 g; 4 months) observed significant reductions in BMI by 1.05 kg/m^2^, fasting blood glucose by 26 mg/dL, triglycerides by 30 mg/dL, and total cholesterol by 6.7 mg/dL (all *p* < 0.05) with whole fruit being an important contributor to adequate total fiber intake [253]. A South African cross-sectional study (627 young adults) observed 97% of the participants consumed <2 g soluble fiber/day, which is suggestive of a possible association between low fruit intake and increased risk for metabolic syndrome [254]. Fruit fiber intake reduces metabolic syndrome risk by a number of mechanisms including: (1) reducing the risk of central obesity, insulin resistance, dyslipidemia, and systemic inflammation; and (2) promoting colonic microbiota health and diversity associated with higher SCFAs levels, reduced risk of low-grade systemic inflammation and metabolic endotoxemia [255].

### 3.6. Cancer

Excess body weight, sedentary lifestyles, unhealthy dietary habits such as low fiber, fruits and vegetables and high meat intake can increase cancer risk [256]. The American Cancer Society recommends eating >2.5 cups/day of fruits and vegetables for cancer prevention [257].

#### 3.6.1. Colorectal Cancer

**Figure 18:**
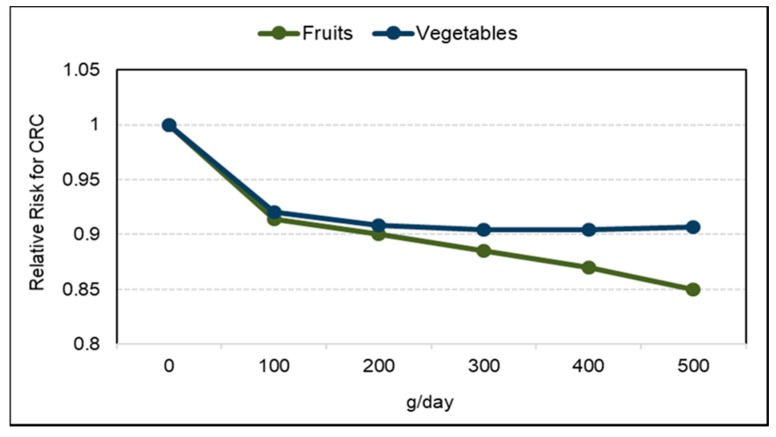
Non-linear dose-response effect of increasing intake of fruits and vegetables on colorectal cancer (CRC) risk from meta-analysis of cohort studies [260].

Colorectal cancer (CRC) is one of the most commonly diagnosed cancers in the world and a leading cause of cancer death, despite major advances in screening, surgery and oncology. The 2007 and 2011 World Cancer Research Fund (WCRF) and the American Institute for Cancer Research (AICR) reports showed convincing meta-analyses evidence that fiber, fruit and vegetables are inversely associated with CRC risk [258,259]. A 2011 meta-analysis (19 cohort studies; 1.7 million subjects) found an inverse association between intake of fruits and vegetables and CRC risk with most of the risk reduction observed at about 100 g/d (Figure 18) [260]. An EPIC prospective cohort study (>500,000 participants) observed that the risk of CRC was inversely associated with intakes of total fruits and vegetables, and total fiber [261]. The Shanghai Men’s Health Study (61,274 men) observed that increased fruit intake was significantly inversely associated with CRC risk, whereas vegetable intake was not associated with CRC risk [262]. A systematic review and meta-analysis observed a lower risk of CRC for whole grains by 5% per 30 g/d, fruits and vegetables by 3% per 100 g/d, dairy by 7% per 200 g/d and an increased CRC risk for red meat by 12% per 100 g/d and for processed meat by 17% per 50 g/d [263]. A meta-analysis (2 cohort and 5 case-control studies) estimated that higher apple intake reduced CRC risk by 28% (*p =* 0.001) [264]. The Malmö Diet and Cancer Study (27,931 participants) observed high intakes of total fiber, and fruits and berries significantly lowered risk for colon cancer by approximately 30% compared to no lowering effects for vegetables and fiber rich cereals [265], which is consistent with a 2018 systematic review and meta-analysis (25 observational studies) observation that higher total fiber intake reduced colon cancer risk by 26% (*p =* 0.000) [266]. A meta-analysis of 20 observational studies [267] and another meta-analysis of 5 cohort and 17 case-control studies [268] showed that each 10-g fruit fiber and high fruit intake reduced adenoma risk by 21% compared to a reduced risk of 9% for vegetables. Secondary analyses of the Polyp Prevention Trial found that super dietary compliers consuming 12 g fiber and 3 fruit and vegetable servings/1000 kcals significantly reduced adenoma recurrence risk by 32% and lowered multiple and/or advanced adenoma recurrence risk by 50% compared to less compliant controls (*p* < 0.05) after 4 years [269]. The Prostate, Lung, Colorectal and Ovarian Cancer Screening Trial (57,774 subjects; 12 years of follow-up) found that higher fruit and vegetable intake significantly reduced the risk of multiple adenomas by 39% and risk of CRC in individuals with high processed meat intakes by 26% [270]. Pooled data from the Nurses’ Health Study and Health Professionals Follow-up Study (1575 participants) observed lower multivariate adjusted CRC specific mortality risk per 5-g increment for fruit fiber by 9% lower risk (*p =* 0.58), vegetable fiber by 18% (*p =* 0.22), and cereal fiber by 33% (*p =* 0.007) [271]. Fiber is the primary dietary component of whole plant foods associated with promoting a healthy colonic microbiota which aids in reducing tumorigenic inflammation, carcinogen production, and altered cellular responses in susceptible individuals [272].

#### 3.6.2. Lung Cancer

Lung cancer is the leading cause of cancer death worldwide with approximately 2 million global deaths attributed to cigarette smoking [273]. Tobacco use is the leading cause of lung cancer resulting in 55% of lung cancer deaths in women and over 70% of lung cancer deaths in men. The current evidence from prospective studies suggest a potential protective role for fruit in lung cancer etiology. A 2015 dose-response meta-analysis of cohort studies (19 studies; approx. 2 million participants) found that each one serving of fruits reduced lung cancer risk by 5% compared to a 3% reduction for each serving of vegetables [274]. Another 2015 meta-analysis of fruit (38 observational studies; 20,213 lung cancer cases) showed that increased fruit intake lowered lung cancer risk by 20% compared to lower fruit intake [275]. The associations with fruit and lower lung cancer risk were stronger for women than for men. A 2016 systematic review and meta-analysis (18 prospective studies) found that high fruit intake reduced lung cancer risk by 18% with each 100-g intake lowering risk by 8% up to 400-g/day [276]. These risk reductions were significant in current smokers but not in former or never smokers. A 2014 EPIC prospective cohort study (>500,000 participants; 10 European countries) observed that the risk of lung cancer was inversely associated with fruit intake but was not associated with vegetable intake; this association with fruit intake was restricted to smokers [261]. In a 2016 meta-analysis (3 case-controlled and 7 cohort studies) the high intake of apples was found to significantly lower lung cancer risk by 25% in case-control studies (*p =* 0.001) and 11% in cohort studies (*p* < 0.001) compared to low intake [264]. The Black Women’s Health Study (47,000 women) observed an insignificant 14% lower lung cancer risk for high vs. low fruit and vegetable intake, regardless of smoking history, but the overall fruit and vegetable intake was relatively low in this study population [277].

### 3.7. Successful Aging

**Figure 19:**
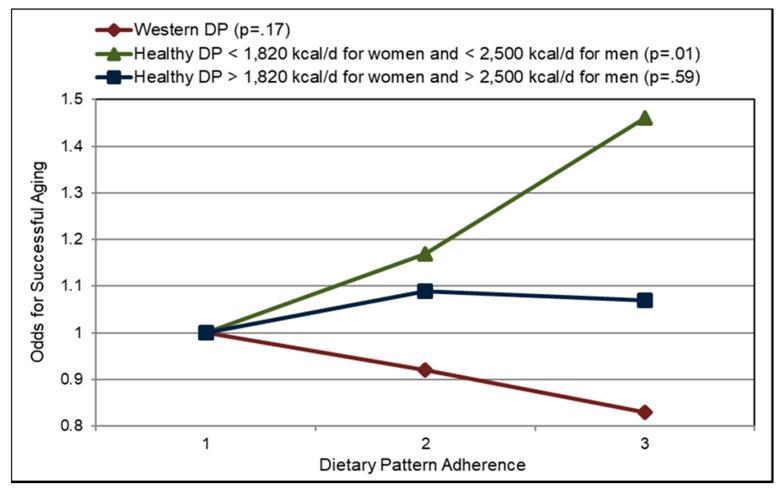
Effects of a healthy dietary pattern (DP) stratified by energy intake level, and the Western DP on the odds for successful aging (multivariate adjusted) [279].

Successful aging is associated with being free of excessive progressive deterioration of physical and mental functions and chronic disease (obesity, coronary artery disease, stroke, diabetes, and cancer); having good mental, physical and respiratory health, and functional independence [278]. Health issues such as increased prevalence of chronic disease and risk of premature death increase substantially beginning in middle-age, but an estimated 80% of health problems in older age could be prevented or delayed by healthy lifestyle changes. A French cohort study (2796 middle aged; 13 years) observed that a higher adherence to: (1) a Western dietary pattern reduced the odds for successful aging by 17%; (2) a healthy high energy dietary pattern improved odds for successful aging by 7%; and (3) a healthy moderate energy dietary pattern improved odds for successful aging by 46% (Figure 19) [279].

#### 3.7.1. Fruit and Fiber

**Figure 20:**
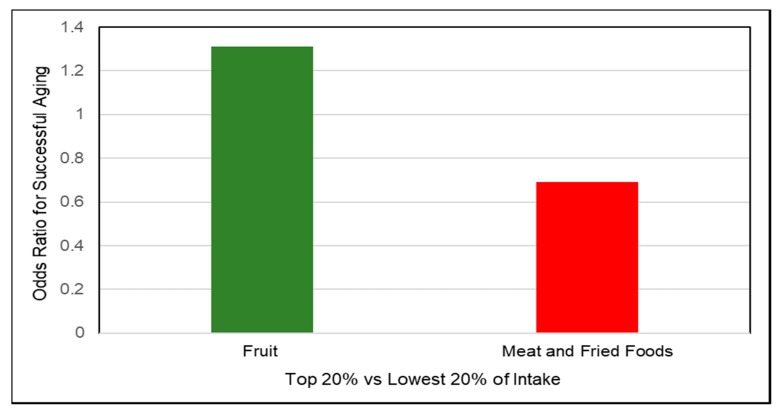
Effect of key dietary pattern food items associated with the odds of successful aging (all *p*-trend across quintile groups = 0.001) [281].

**Figure 21:**
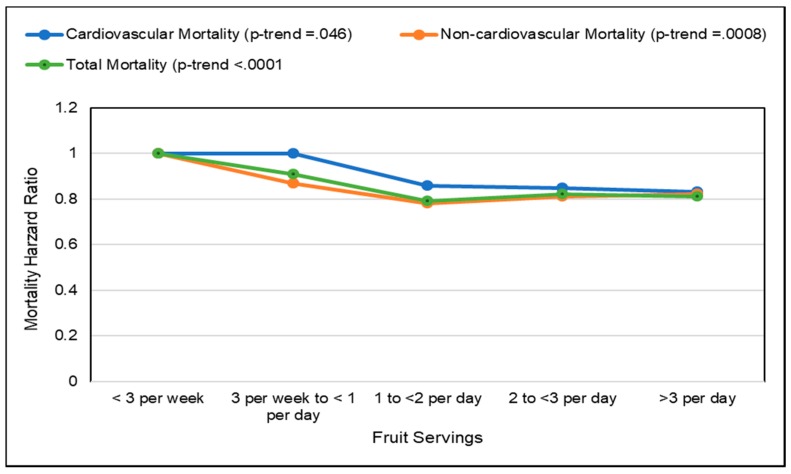
Association of fruit intake with mortality risk for a composite of European, American, Japanese and Chinese adults (mean age 50 years over 7.4 years; multivariate adjusted) [4].

Whole fruits are among the best low energy dense and fiber-rich healthy food options. A UK EPIC-Norfolk study cross-sectional analysis (16,792 adults; mean age 62 years) showed that each daily intake of two servings of fruits and vegetables increased the odds of good functional health by 11% [280]. An Australian prospective study (6308 healthy adults at baseline; 11.7 years) observed that the likelihood of successful aging for dietary patterns rich in fruit was improved by 30% and odds reduced by 30% for patterns rich in meat and fried foods (Figure 20) [281]. The (Australian) Blue Mountains Eye Study (1609 participants; mean age 61; 10 years) observed that a higher dietary nutrient quality score was associated with better odds of successful aging by 58% [282]. Participants with the highest intake of fruit had 49% better odds for successful aging and lower risk of premature death by 23% compared to those with low fruit intake. Successful aging odds improved by 2% for each 1-g increase in total fiber intake and those consistently consuming fruit fiber below the median intake were 36% less likely to age successfully compared to those above the median intake of fruit fiber [283]. A meta-analysis (20 cohort studies; 760,629 participants) found a linear dose-response relationship with reduced stroke risk for fruits by 32% and for vegetables by 11% per 200 g/day with citrus fruits, apples, pears, and green leafy vegetables being the most effective in reducing stroke risk [284]. A 2017 systematic review and dose-response meta-analysis (94 cohort studies; 2,123,415 participants) found significantly reduced all-cause mortality risk by 15% per 200 g total fruit intake/day [285]. Specific fruits varied in their effects on lowering odds of all-cause mortality per 100 g intake; for apples and pears by 20%, bananas by 5%, berries by 15%, and citrus fruit by 6%. Prevencion con Dieta Mediterranea (PREDIMED) trial (7216 older adults with high CVD risk; 9 years) showed that a person who consumed >210 g fruit/day or had fiber intake of >20 g/day had significantly lower risk of premature death by about 40% for each [286]. The UK Women’s Cohort Study found that women consuming 7 portions of fruit/day had a 43% lower risk of death from CVD compared with women consuming 2.5 portions/day (*p =* 0.013) [163]. A cross-sectional study (1000 middle age adults; 12 weeks) observed that higher fruit intake significantly reduced BMI and inflammatory biomarkers, which are associated with increased odds of successful aging (Table 3) [287]. A cross-sectional study (600 postmenopausal women) observed a significant association between reduced urinary metabolite prostaglandin E2 (PGE2), an inflammatory mediator that plays key roles in promoting tumor development and progression, and increased consumption of fruits (*p*-trend = 0.02) [288]. Microbiota SFCAs can act as anti-inflammatory mediators by regulating PGE-2 and cytokines. The Prospective Urban Rural Epidemiology [PURE] study (135,335 adults from Europe, US, Japan and China; 7.4 years) observed that fruit intake was associated with lower risk of cardiovascular, non-cardiovascular, and total mortality (Figure 21) [4]. A 2018 umbrella review (18 meta-analyses including a total of 298 prospective observational studies) found that a higher fiber intake was convincingly associated with decreased odds of all-cause and CVD mortality [3]. Suggestive evidence was also found for associations with lower risk of stroke, type 2 diabetes and several cancers (i.e., pancreatic, gastric, esophageal, colon, endometrial, breast, and renal). A Canadian cost-of-illness analysis estimated that each additional 1 g fiber/day resulted in an annual $3 to 51 million in savings in type 2 diabetes care and $5 to 92 million in cardiovascular disease care [289].

#### 3.7.2. Mechanisms

Aging is a complex process at the cellular level involving genetic changes that cause a progressive loss of physical capabilities [290]. Many of the factors that control aging are related to gene expression including epigenetic and telomeric changes that are outside of the information encoded in the DNA. Lifestyle factors such as diet, exercise, and stress can modify cell function and change epigenetic and telomere information that affect gene expression and nucleic stability. Long telomeres in old age help to maintain key cellular processes that reduce the number of years a person experiences poor health during aging. A 2018 NHANES analysis (5674 adults) found that fiber intake of 10 g per 1000 kcal increased leukocyte telomere length by 67 base pairs (*p =* 0.01), which reduced biologic (cellular) aging by 4.3 years compared to the low fiber Western diets of 6.6 g fiber per 1000 kcal [291]. Two longitudinal studies (521 subjects; 5 years) found that longer telomere length was significantly predictive of lower body weight, BMI, waist circumference, and dietary inflammatory index, and was associated with the higher fruit and fiber intake associated with high adherence to the Mediterranean diet [292,293]. The Framingham Heart Study Offspring cohort study observed that the intake of whole fruit up-regulated epigenetic gene function for immunosurveillance, and chromosome and telomere maintenance pathways, attributed to the effects of fruit fiber on colonic microbiota health [294]. Emerging fiber mechanisms associated with successful aging include: (1) enhancing the colonic probiotic microflora profile and production of SCFAs; (2) improving the colonic barrier function to protect against clinical inflammation; (3) increasing colonic peptides that are important in glucose and insulin homeostasis and lipid metabolism; and (4) mimicking many of the effects of caloric restriction including upregulation of genes involved in energy metabolism [295].

### 3.8. Lung Function

Impaired lung function related to allergies, asthma, environmental factors and tobacco usage is a major worldwide health issue affecting 100 s of millions of children and adults.

#### 3.8.1. Asthma Severity and Wheezing

Asthma, a chronic respiratory inflammatory condition, represents a major public health burden worldwide. It affects about 300 million people of all ages and is increasing in prevalence [296,297]. Symptoms of asthma in low- and middle-income countries can be as high as rates found in more developed countries. Among children aged 5–14 years, asthma is among the top ten global ranking conditions for disability-adjusted life years. Growing evidence from observational studies indicates that increased whole fruit intake is associated with reduced severity of asthma symptoms such as airway constriction, inflammation, bronchial hyper-responsiveness, coughing, wheezing, and chest tightness. A 2017 meta-analysis of fruit and vegetable intake on risk of asthma/wheeze and immune responses (30 cross-sectional, 13 cohort, 8 case-control and 7 experimental studies; children and adults) found no significant association between fruit intake and risk of prevalence of asthma but the intake of fruit was inversely associated with the severity of asthma in secondary prevention studies, with a higher fruit intake lowering severity by 39% [297]. Fruit intake was also negatively associated with risk of wheezing. Vegetable intake was not associated with the severity of asthma or wheezing. A systematic review by the European Academy of Allergy and Clinical Immunology recommended an increased intake of fresh fruits as a way of reducing the number and severity of asthma flare-ups, particularly in children [298]. The Prevention and Incidence of Asthma and Mite Allergy study (2870 children; followed from birth to 8 years of age) observed that each day of fresh fruit consumption per week at 2–3 years of age was associated with reduced asthma symptoms and atopy by 7% at age 8 years, but no asthma protection was observed for cooked vegetables [299]. Long-term fruit intake was inversely associated with asthma symptoms and sensitization to inhaled allergens with 10% reduction for each daily serving. A Brazilian case-controlled study (171 children; 30 days) observed that regular consumption of fruits was associated with an 81% lower risk of asthma severity or persistence [300]. A Portuguese cross-sectional study (174 asthmatics) observed that higher fresh fruit intake significantly decreased risk of uncontrolled asthma by 71% (*p =* 0.015) [301]. Also, high adherence to the Mediterranean diet reduced the risk of uncontrolled asthma by 78%, after adjusting for gender, age, education, inhaled corticosteroids and energy intake (*p*-trend = 0.028). In children, a 2014 systematic review and meta-analysis (1 cohort study and 13 cross-sectional studies) found higher fruit intake significantly reduced wheezing in children ≤11 years old by 17%, and for children >11 years old by 19% and reduced asthma severity in children >11 years old by 24%, but not for children ≤11 years old who showed an insignificant reduced risk of 11% [302]. In adults, a meta-analysis (3 cohorts, 2 case-controls, and 4 cross-sectional studies) found that higher intake of fruit reduced the overall risk of asthma severity by 23% compared with the lowest intake. In subgroup analysis, increased intake of apples or citrus fruit reduced the risk of asthma severity by about 24%. A 2016 RCT (90 adults with uncontrolled asthma; fruit and vegetable rich DASH diet vs. usual-care control; 6 months) found that the DASH diet modestly but significantly improved overall asthma control score by 19% (*p =* 0.04) and quality of life score by 39% (*p =* 0.004) compared to the subjects’ usual diet [303]. The 2017 (Latin American) International Study of Asthma and Allergies in Childhood Phase III (143,967 children with asthma; 11 Latin American countries) observed in children 6 to 7 years of age that adequate fruit intake can reduce wheezing risk by up to 35% compared to low fruit intake [304]. For 13 to 14-year-old children, there were similar but slightly attenuated wheezing lowering effects for increased fruit intake. A 2018 meta-analysis showed that higher fruit intake was associated with increased colonic microbiota SCFAs levels which suppressed asthma airway restrictions by helping to lower levels of CRP, TNF-*α,* and mast cell, and increasing *γ δ*-T cell and T_reg_ cell numbers and function (*p* < 0.05) [305]. Low fruit (e.g., fiber and polyphenols) can increase microbiota dysbiosis, which is associated with increased risk of asthma severity [303,304,305]. Individuals with severe persistent asthma often have a high adherence to the Western diet and consume significantly less fruit fiber compared to healthy controls, which is partly associated with the increased colonic microbiota dysbiosis and lung axis effects associated with increased airway inflammation and adverse immune response reducing breathing capacity of the lungs [306,307,308]. A meta-analysis (2 RCTs; 249 children; age range 2–5 years) found that prebiotic fiber reduced the risk of asthma or wheezing by 63% when compared to the control group [309]. Adequate intake of whole fruits is associated with reduced severity of asthma in children and adults through fruit fiber effects linked to a healthier colonic microbiome that actively suppresses the severity of airway inflammatory restrictions.

#### 3.8.2. Chronic Obstructive Pulmonary Disease (COPD)

The estimated global number of cases of COPD is approximately 400 million [310]. COPD is multifactorial, and the risk factors include genetic and environmental quality factors (e.g., tobacco smoke, occupational inhalants and air pollutants originating from biomass burning and traffic exhaust) [310,311]. Although tobacco smoking is an established risk factor for COPD, up to 50% of cases of COPD can be attributed to nonsmoking risk factors. Observational studies suggest that fruit intake is positively associated with lung function and inversely related to COPD respiratory symptoms and death and more effective than increased vegetable and whole-grain intake [312]. A cross-sectional analysis of the Atherosclerosis Risk in Communities Study (15,792 adults) observed that high adherence to a prudent diet rich in fruits and vegetables was associated with a lower prevalence of COPD by 18%, coughing by 23%, and a higher forced expiratory volume compared to 62% increased prevalence of COPD, a higher level of wheezing, coughing and phlegm by 27 to 37% and a reduced forced expiratory volume for high adherence to the Western diet [313]. A 2017 Cohort of Swedish Men study (44,335 men; 13.2 years) observed a strong inverse association for total fruit and vegetable intake and COPD in smokers but not in never smokers (*p*-interaction = 0.02) [314]. The risk of COPD was significantly reduced in current smokers by 8% and in ex-smokers by 4% for each one serving/day of total fruit and/or vegetables. A Swedish Mammography Cohort (34,739 women; age range 48–83 years; 12-year follow-up) observed that women consuming 2.5 or more fruit servings/day had a 37% lower risk of COPD (*p*-trend < 0.0001) compared to those consuming <0.8 serving/day [315]. Long-term vegetable intake was not associated with lower COPD risk. In current and ex-smokers, women with low fruit intake (<1 serving/day) had a 38-fold and 13-fold higher COPD risk, respectively, than those consuming 3 or more fruit servings/day. A 2016 NHANES analysis (1921 adults) observed that participants with higher fiber intake had healthier mean forced expiratory volume by 82 mL/s and forced vital capacity by 129 mL/s, than those with lower fiber intake (*p =* 0.05 and 0.01), which is consistent with the consumption of higher vs. lower fruit intake [316]. The 2018 Cohort of Swedish Men study (45,058 men w; 13.1 years) observed an inverse association between total fiber intake (≥ 37 g/day vs. <24 g/day) and COPD in smokers by 46% and ex-smokers by 38% [317]. Never smokers had a 7% reduction in COPD with higher fiber intake (*p* interaction = 0.04). Fruit fiber reduced COPD risk in smokers by 35% (*p*-trend = 0.001) and in ex-smokers by 23% (*p*-trend = 0.17). The development or progression of COPD is associated with an over-active immune system characterized by increased neutrophil and macrophage activation [318]. Adequate fruit fiber intake promotes healthy lung immune function via the colonic microbiota-liver-lung axis, which affects systemic inflammatory cytokines and immune mediators (notably, IL-6 and CRP). 

### 3.9. Psychological Well-Being

The consumption of low fiber Western diets can increase the risk of microbiota dysbiosis and associated reductions in psychological well-being, which in turn can induce poor stress response behavior and moods through the vagus nerve, whereas fiber rich healthy diets can improve microbiota health to positively impact psychological well-being [319,320,321,322,323]. SCFAs such as butyrate and propionate directly affect brain physiology and behavior by acting on microglial cells and astrocytes to promote anti-inflammatory action and manage overall brain maintenance by scavenging for damaged or unnecessary neurons and synapses, and infectious agents.

#### 3.9.1. Children

Emerging observational studies suggest that increased fruit intake may improve psychological well-being. The Canadian CHILD-Edmonton sub-cohort study (688 mothers and infants) observed that each maternal daily serving of fruit consumed during pregnancy was associated with a 2.4-point increase in a child’s 1-year cognitive development and 0.67-point increase in 1-year adaptive development (*p =* 0.05; adjusted for maternal education and vitamin supplementation, socioeconomic status, and other related factors) [324]. The Australian Victorian Child’s Health and Well-being study (3370 children) observed that children with a higher number of daily fruit servings had significantly better odds of emotional control, and problem management and relationship building skills (pro-social behavior) [325]. The Western Australian Pregnancy Cohort (Raine) Study (1324 adolescents) observed that withdrawal/depression and delinquent/aggressive behaviors were significantly associated with Western diets and prosocial/positive behaviors were significantly associated with higher intake of fresh fruits and green leafy vegetables (components of a healthy diet) [326].

#### 3.9.2. Adults

**Figure 22:**
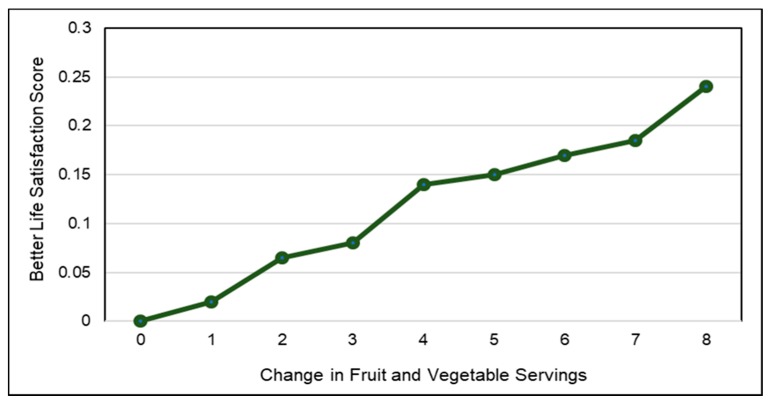
Association between the number of servings of fruits and vegetables/day and increases in life satisfaction (*p* < 0.001) [329].

In adults, emerging evidence suggests that higher daily intake of fiber-rich fruit and vegetable servings is associated with lower incidences of anxiety, greater happiness, higher life satisfaction, and greater social-emotional well-being. A cross-sectional study observed that adults consuming high quality diets had 67% better psychological well-being relative to those with low quality diets (*p* = 0.007) [327]. Of the food groups, this improvement in well-being was only significant for higher servings of fruits and vegetables. A New Zealand and US on-line cross-sectional study observed that the consumption of raw fruits and vegetables had a stronger correlation with psychological well-being (positive mood) than the consumption of cooked or canned (processed) fruits and vegetables [328]. A 2016 Household, Income, and Labour Dynamics in Australia Survey longitudinal study observed that increased fruit and vegetable intake was predictive of increased happiness, life satisfaction, and well-being [329]. For 8 servings per day, there was up to 0.24 life-satisfaction points, which is equal in size to the psychological gain of going from unemployment to employment (Figure 22). A longitudinal study in young adults observed that eating adequate fruits and vegetables was associated with improved indicators of flourishing including greater well-being, creativity, and curiosity, which can play an important role in psychological resilience [330]. Another longitudinal study in young adults observed that those consuming more fruits and vegetables had higher scores in openness and extraversion, and to some extent conscientiousness, than their lower fruit and vegetable consuming peers [331]. A parallel RCT of low fruit and vegetable consuming young adults found that providing 2 added daily servings of fruits and vegetables to eat significantly improved psychological well-being (e.g., feelings of vitality, flourishing, and motivation) compared to just reminding them to eat their fruits and vegetables [332].

### 3.10. Depression

**Figure 23:**
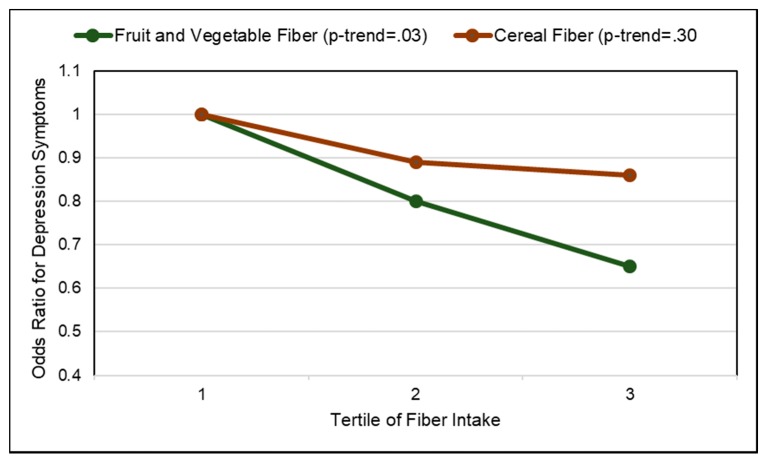
Odds ratio for depressive symptoms with fruit and vegetable fiber intake compared to cereal fiber (fruit and vegetable fiber tertiles = 1.5 g, 2.5 g and 4.0 g) [336].

Chronic depression progressively worsens a person’s state of health, which makes it one of the largest contributors to global disability and suicide deaths [333,334]. Emerging observational studies suggest that higher fruit and fiber intake are associated with lower risk of depression, anxiety, and high psychological distress. An Iranian cross-sectional study found that women in the highest quintile of fruit intake (≥464 g fruit/day) had significantly lower odds of depression by 57%, anxiety by 50%, and psychological distress by 60% compared to those with the lowest intake of fruit (<144 g fruit/day) [333]. A 2018 systematic review and meta-analysis (6 cohort studies) found that the pooled risk for depression was reduced by 17% for the highest vs. the lowest category of fruit intake and a dose response analysis (3 cohort studies) showed a 3% lower risk of depression for every 100 g of fruit consumed [334]. A nationally representative longitudinal study (>6000 Canadian adults; 2 years) observed that increased intake of fruits and vegetables at any timepoint was associated with decreased distress, and lower risk of depression [335]. The associations between fruit and vegetable consumption and reduction of mental distress and depression can be further modified by a person’s level of social support, physical exercise and smoking habits, especially at the extremes. The Furukawa Nutrition and Health Study (1977 employees) found that fiber from fruits and vegetables was 21% more effective in reducing depression symptoms or improving mood than cereal fiber (Figure 23) [336]. A NHANES study of 2007 to 2014 data (16,807 adults) observed that total, vegetable, and fruit fiber intakes were significantly associated with an approximately 40% lower risk of depressive symptoms, whereas the association of cereal fiber intake with depressive symptoms was insignificant with a 10% lower risk [337]. In dose-response analyses, the daily intake of fruit or vegetable fiber was associated with a 30% reduction in the risk of depression. The 2017 English Longitudinal Study of Ageing observed that conscientiousness (a personality trait of being thorough, efficient and organized with a desire to do a task well) was associated individuals being physically active, having lower body weight, higher fruit and vegetable intake and a reduced risk of depression (all *p* < 0.001) [338]. The evidence of adequate intake of fruits and vegetables and their fiber components for reducing the risk of depression or helping manage depressive symptoms is limited but consistent.

### 3.11. Bone Mineral Density

Adequate intake of whole fruit and fruit fiber has been associated with increased bone mineral density, especially the attainment of peak bone mass during adolescence and minimized bone resorption among postmenopausal women [339]. Between 25 and 40 years of age, women are still gaining or stabilizing bone mass in all skeletal sites, whereas the bone density among men, particularly in the hips, is declining [340]. Accelerated loss among women during the perimenopausal period occurs between ages approximately 40 to 54 years, and then stabilizes. This accelerated loss is a major determinant of the differences in the patterns of bone loss between women and men. A second period of accelerated bone loss begins at age 70 among women, notably in the total hip, which is substantial by age 75 years and is associated with the increased incidence of hip fractures among elderly patients. Diets rich in prebiotic fiber such as fruit pectin can improve calcium absorption and inhibit osteoclast bone resorption, while maintaining osteoblast bone formation activity [341]. Specifically, three mechanisms are occurring: (1) calcium is an important component of fruit cell walls for structure and function and the fermentation of fiber in the colon releases bound calcium for absorption; (2) fiber fermentation SCFA metabolites propionate and butyrate induce metabolic reprogramming of osteoclasts by downregulating critical osteoclast genes; and (3) increased SCFAs lower colonic pH to enhance calcium absorption.

#### 3.11.1. Observational Studies

Several observational studies support the benefits of increased fruit intake on bone mineral density, especially in adolescent children and older postmenopausal women. A 2014 Northern Ireland cross-sectional study observed that 12-year old girls consuming high amounts of fruit had significantly improved heel bone mineral density compared to the lower fruit consumers (lower fruit intake was defined as ≤83 g/day and high fruit intake as >200 g/day) [342]. A UK cross-sectional study observed in adolescent boys and girls and older women that fruit intake was positively associated with spine bone mineral content, after adjusting for body size and type, however, no significant positive association was shown for the intake of vegetables [343]. No significant associations were found for young women or older men. A 2018 Framingham Offspring Study (792 men, mean age 58 years; 1065 women; mean age 57 years; 8 years follow-up) observed significantly reduced annualized loss of femoral neck bone mineral density (predictive of hip fracture risk) per 5 g/day total fiber by 0.06% or fruit fiber by 0.04% [344]. A 2017 Chinese cross-sectional study (3089 participants; age range 40 to 75 years; 66% women) observed that high fruit intake had dose-dependent associations with greater bone mineral density and a lower risk of osteoporosis; but this was not shown for vegetables [345]. Higher intake of apples, pears, peaches, pineapples and plums significantly improved the mineral density for whole body and spine, total hip and femoral neck bone regions by approximately 0.014 g/cm^3^/year. Also, an increased intake of fruits and vegetables from 349 to 685 g/day reduced the risk of an osteoporotic fracture 8.3%. A 2012 Chinese cross-sectional study (adolescent boys and girls, young women and postmenopausal women) observed that there was a significant positive association between higher fruit intake and bone mineral density for all participants combined (*p*-trend <0.001 to 0.002) in total body and lumbar spine, total hip and femoral neck regions vs. lower fruit intake [346].

#### 3.11.2. RCTs

Several RCTs support the bone protective effects of increased dried plum intake in postmenopausal women by slowing rate of bone resorption. A parallel RCT (160 post-menopausal women; mean age 57 years; 100 g/d dried plums or dried apples plus 500 mg calcium plus 400 IU vitamin D daily; 12 months) found that both plums and apples increased bone mineral density from baseline in forearm ulna, spine, femoral neck, total hip and whole-body and plums promoted significantly more bone mineral density of forearm ulna and spine than dried apples [347]. A dose-response RCT (48 osteopenic women; age 65–79 years old; 50 g and 100 g of dried plums vs. control; 6-months) showed that both dried plum doses significantly increased total body bone mineral density resulting in a higher bone-specific alkaline phosphatase: tartrate-resistant acid phosphatase ratio compared to no change in the control group (*p* < 0.05) [348].

### 3.12. Seborrheic Dermatitis

Seborrheic dermatitis is a common chronic relapsing skin condition that causes redness, scaly patches, and dandruff, which most often affects the scalp, but it can also develop in oily areas of the body, such as the face, upper chest, and back [349]. A 2018 Dutch cross-sectional study observed that high adherence to the Western diet increased seborrheic dermatitis risk by 34% (*p*-trend = 0.07] [349]. For women, a higher adherence to the Western diet was associated with an increased risk of seborrheic dermatitis by 47% (*p =* 0.03) but there was no significant association between the Western pattern and seborrheic dermatitis in men (increased risk of 18%; *p =* 0.67). Increased fruit intake (highest vs. lowest quartile) reduced seborrheic dermatitis risk by 25% (*p =* 0.03) but increased vegetable intake had no effects on risk. This study did not find an association between increased antioxidant intake (FRAP-score) and seborrheic dermatitis with a 6% reduced risk (*p*-trend = 0.88). Since colonic microbiota dysbiosis has been associated with chronic inflammatory skin disorders, such as atopic dermatitis and psoriasis, the beneficial effect of increased fruit intake on seborrheic dermatitis may be partially due to the prebiotic effects of fruit fibers such as pectin, which promote a healthy colonic microbiota associated with lower systemic inflammation and better immune function [350].

### 3.13. Autism Spectrum Disorder (ASD)

Children with ASD are often picky eaters with low intake of fiber-rich foods, including fruits and vegetables and have an increased prevalence of gastrointestinal abnormality and microbial dysbiosis. This can interfere with gut-brain axis communications, which may exacerbate ASD behavior traits [351,352]. Increased fruit fiber such as pectin intake has been suggested to correct gastrointestinal abnormality and promote microbial health to potentially enhance gut-brain communication and potentially reduce the ASD symptoms. Diets of children with ASD often consist of fewer than 20 foods, repetitive eating patterns, and nutrient inadequacies, which can lead to increased colonic microbiota dysbiosis, gastrointestinal distress, flatulence and abdominal pain and lower intakes of fiber such as pectin and vitamin C, suggesting low fruit intake [353,354]. A 2018 intervention trial (41 autistic children; 75% male; mean age 8 years old; 6 weeks) showed that supplementation of the diet with a prebiotic resulted in significant improvement in anti-social behavior, and gastrointestinal and microbiota health relative to baseline diets [355]. A recent case report, described a 4-year old boy with ASD who developed scurvy secondary to selectively eating a diet very low in fruits and vegetables [356]. Larger studies are needed to investigate whether prebiotics such as increased fruit intake may have beneficial effects on ASD symptoms.

## Figures and Tables

**Table 1:** Specific whole fruit fiber, available carbohydrates and energy content [1,2,6,7,8,9].

Fruit Source	Serving Size *	Estimated Fiber Components				Sugar (Starch) (100-g)	Energy Density (kcal/g)	Energy (kcal) 100-g
		Total (100-g)	Insoluble (100-g)	Soluble (100-g)	Pectin ** (100-g)			
**Fresh Fruit**								
Apples	1 (182 g)	2.4	1.7	0.5	0.8	10	0.5	52
Avocados	1/3 (50 g)	6.8	4.4	2.4	2.4	< 1.0	1.6	160
Bananas	1 (118 g)	2.6	1.8	0.8	0.6	12 (5)	0.9	89
Blackberries	1 cup(144 g)	5.3	4.7	0.6	1.4	4.9	0.4	43
Blueberries	1 cup(148 g)	2.8	2.4	0.3	0.8	10	0.6	57
Cherries	1 cup(138 g)	2.2	1.6	0.6	0.7	13	0.6	63
Figs	1 (100 g)	3.0	2.4	0.6	1.0	16	0.7	74
Grapefruits	1 cup(154 g)	1.6	1.1	0.5	0.6	7.0	0.4	42
Guavas	1 cup(165 g)	5.4	4.2	1.8	1.5	9.0	0.7	68
Kiwis	2 (138 g)	3.0	2.2	0.9	0.7	9.0	0.6	61
Mangoes	1 cup(165 g)	1.6	1.0	0.6	0.5	14	0.6	60
Oranges	1 (131 g)	2.4	1.4	1.0	0.8	9.1	0.5	47
Papayas	1 cup(145 g)	1.7	1.4	0.3	0.5	7.6	0.4	43
Pears	1 (166 g)	3.1	2.2	0.9	1.0	10	0.6	60
Plantains	½ (134 g)	2.2	1.5	0.7	0.4	2.2 (30)	1.5	149
Pomegranate arils	1 cup(122 g)	5.7	4.1	1.6	2.0	12	0.8	81
Raspberries	1 cup(123 g)	6.5	5.3	1.2	1.6	4.4	0.5	52
Strawberries	1 cup(152 g)	2.0	1.5	0.5	0.7	4.7	0.3	33
Apricots	2 (70 g)	2.0	1.0	1.0	0.7	9.1	0.5	49
Cantaloupes	1 cup(177 g)	0.9	0.6	0.3	0.3	7.9	0.3	34
Green grapes	1 cup (92 g)	0.9	0.6	0.3	0.2	17	0.7	69
Peaches	1 (150 g)	1.5	0.9	0.6	0.5	9.0	0.4	39
Pineapples	1 cup(165 g)	1.4	1.1	0.3	0.5	10	0.5	50
Plums	2 (132 g)	1.4	0.9	0.5	0.4	11	0.5	45
Watermelons	1 wedge (286 g)	0.4	0.3	0.1	0.1	6.3	0.3	29
**Dried Fruit**								
Apricots	6 (40 g)	10	7.5	2.5	3.0	37	2.6	258
Cranberries	¼ cup(40 g)	7.5	5.0	2.5	2.5	72	3.3	325
Dates, pitted	5–6 (40 g)	7.5	6.0	1.5	2.5	72	3.0	300
Dried Figs	1/3 cup (40 g)	12	8.8	37	4.3	50	2.8	275
Prunes	7 (40 g)	7.5	5.0	2.5	2.5	37	2.5	250
Raisins	¼ cu(40 g)	5.0	3.7	1.2	1.7	73	3.0	300

* Based on commonly consumed fruit servings from USDA Food Composition Databases [7] ** Fruit pectin = mean 35% (range 20–40%) of total fiber [9].

**Table 2:** Mean change in cardiovascular disease (CVD) risk factors for newly diagnosed CVD patients after a 4-week prevention diet primarily of fruits, vegetables, avocados and seeds [164].

Variable	Baseline Value	4-Week Value	% Change	*p*-Value
Total cholesterol (mg/dL)	217	183	−34	<0.0005
LDL-C (mg/dL)	143	118	−25	<0.0005
HDL-C (mg/dL)	55	105	−5	<0.0005
Triglycerides (mg/dL)	124	105	−20	=0.008
Insulin (µIU/mL)	14.6	10.3	−4.3	<0.0005
Glucose (mg/dL)	90	87	−2.9	=0.25
HbA1c (%)	5.9	5.7	−0.2	=0.002
hs-CRP (mg/L)	7.8	5.3	−2.5	=0.001
Weight (kg)	108	101	−7.0	<0.0005
BMI (kg/m^2^)	37.5	35.2	−2.3	<0.0005
Diastolic BP (mm Hg)	91	82	−9.0	<0.0005
Systolic BP (mm Hg)	147	130	−17	<0.0005
Energy (kcal/day)	2053	1369	−684	<0.0005
Dietary Fiber (g/day)	20	51	+31	<0.0005

**Table 3:** Servings of fruit and BMI and inflammatory biomarkers in US adults [287].

Variables	<2 Fruit Servings/Day	2 Fruit Servings/Day	>2 Fruit Servings/Day	*p*-Trend
BMI (kg/m^2^)	27.9 ± 5.8	26.9 ± 5.8	25.9 ± 5.0	<0.0001
CRP (mg/L)	1.80 (1.55–2.08)	1.42 (1.26–1.62)	1.16 (1.02–1.33)	<0.0032
IL-6 (pg/mL)	1.81 (1.69–1.94)	1.59 (1.50–1.69)	1.34 (1.26–1.42)	<0.0001
TNF-α (pg/mL)	1.95 (1.79–2.13)	1.67 (1.54–1.80)	1.46 (1.35–1.58)	<0.0001
F_2_-isoprostanes (pg/mL)	43.9 (42.3–45.4)	40.9 (39.6–42.1)	36.8 (35.6–37.9)	<0.0001
